# The global biogeography of tree leaf form and habit

**DOI:** 10.1038/s41477-023-01543-5

**Published:** 2023-10-23

**Authors:** Haozhi Ma, Thomas W. Crowther, Lidong Mo, Daniel S. Maynard, Susanne S. Renner, Johan van den Hoogen, Yibiao Zou, Jingjing Liang, Sergio de-Miguel, Gert-Jan Nabuurs, Peter B. Reich, Ülo Niinemets, Meinrad Abegg, Yves C. Adou Yao, Giorgio Alberti, Angelica M. Almeyda Zambrano, Braulio Vilchez Alvarado, Esteban Alvarez-Dávila, Patricia Alvarez-Loayza, Luciana F. Alves, Christian Ammer, Clara Antón-Fernández, Alejandro Araujo-Murakami, Luzmila Arroyo, Valerio Avitabile, Gerardo A. Aymard, Timothy R. Baker, Radomir Bałazy, Olaf Banki, Jorcely G. Barroso, Meredith L. Bastian, Jean-Francois Bastin, Luca Birigazzi, Philippe Birnbaum, Robert Bitariho, Pascal Boeckx, Frans Bongers, Olivier Bouriaud, Pedro H. S. Brancalion, Susanne Brandl, Francis Q. Brearley, Roel Brienen, Eben N. Broadbent, Helge Bruelheide, Filippo Bussotti, Roberto Cazzolla Gatti, Ricardo G. César, Goran Cesljar, Robin Chazdon, Han Y. H. Chen, Chelsea Chisholm, Hyunkook Cho, Emil Cienciala, Connie Clark, David Clark, Gabriel D. Colletta, David A. Coomes, Fernando Cornejo Valverde, José J. Corral-Rivas, Philip M. Crim, Jonathan R. Cumming, Selvadurai Dayanandan, André L. de Gasper, Mathieu Decuyper, Géraldine Derroire, Ben DeVries, Ilija Djordjevic, Jiri Dolezal, Aurélie Dourdain, Nestor Laurier Engone Obiang, Brian J. Enquist, Teresa J. Eyre, Adandé Belarmain Fandohan, Tom M. Fayle, Ted R. Feldpausch, Leandro V. Ferreira, Leena Finér, Markus Fischer, Christine Fletcher, Jonas Fridman, Lorenzo Frizzera, Javier G. P. Gamarra, Damiano Gianelle, Henry B. Glick, David J. Harris, Andrew Hector, Andreas Hemp, Geerten Hengeveld, Bruno Hérault, John L. Herbohn, Martin Herold, Annika Hillers, Eurídice N. Honorio Coronado, Cang Hui, Thomas T. Ibanez, Iêda Amaral, Nobuo Imai, Andrzej M. Jagodziński, Bogdan Jaroszewicz, Vivian Kvist Johannsen, Carlos A. Joly, Tommaso Jucker, Ilbin Jung, Viktor Karminov, Kuswata Kartawinata, Elizabeth Kearsley, David Kenfack, Deborah K. Kennard, Sebastian Kepfer-Rojas, Gunnar Keppel, Mohammed Latif Khan, Timothy J. Killeen, Hyun Seok Kim, Kanehiro Kitayama, Michael Köhl, Henn Korjus, Florian Kraxner, Dmitry Kucher, Diana Laarmann, Mait Lang, Simon L. Lewis, Huicui Lu, Natalia V. Lukina, Brian S. Maitner, Yadvinder Malhi, Eric Marcon, Beatriz Schwantes Marimon, Ben Hur Marimon-Junior, Andrew R. Marshall, Emanuel H. Martin, Jorge A. Meave, Omar Melo-Cruz, Casimiro Mendoza, Cory Merow, Abel Monteagudo Mendoza, Vanessa S. Moreno, Sharif A. Mukul, Philip Mundhenk, María Guadalupe Nava-Miranda, David Neill, Victor J. Neldner, Radovan V. Nevenic, Michael R. Ngugi, Pascal A. Niklaus, Jacek Oleksyn, Petr Ontikov, Edgar Ortiz-Malavasi, Yude Pan, Alain Paquette, Alexander Parada-Gutierrez, Elena I. Parfenova, Minjee Park, Marc Parren, Narayanaswamy Parthasarathy, Pablo L. Peri, Sebastian Pfautsch, Oliver L. Phillips, Nicolas Picard, Maria Teresa F. Piedade, Daniel Piotto, Nigel C. A. Pitman, Irina Mendoza-Polo, Axel D. Poulsen, John R. Poulsen, Hans Pretzsch, Freddy Ramirez Arevalo, Zorayda Restrepo-Correa, Mirco Rodeghiero, Samir G. Rolim, Anand Roopsind, Francesco Rovero, Ervan Rutishauser, Purabi Saikia, Christian Salas-Eljatib, Philippe Saner, Peter Schall, Mart-Jan Schelhaas, Dmitry Schepaschenko, Michael Scherer-Lorenzen, Bernhard Schmid, Jochen Schöngart, Eric B. Searle, Vladimír Seben, Josep M. Serra-Diaz, Douglas Sheil, Anatoly Z. Shvidenko, Javier E. Silva-Espejo, Marcos Silveira, James Singh, Plinio Sist, Ferry Slik, Bonaventure Sonké, Alexandre F. Souza, Stanislaw Miścicki, Krzysztof J. Stereńczak, Jens-Christian Svenning, Miroslav Svoboda, Ben Swanepoel, Natalia Targhetta, Nadja Tchebakova, Hans ter Steege, Raquel Thomas, Elena Tikhonova, Peter M. Umunay, Vladimir A. Usoltsev, Renato Valencia, Fernando Valladares, Fons van der Plas, Tran Van Do, Michael E. van Nuland, Rodolfo M. Vasquez, Hans Verbeeck, Helder Viana, Alexander C. Vibrans, Simone Vieira, Klaus von Gadow, Hua-Feng Wang, James V. Watson, Gijsbert D. A. Werner, Bertil Westerlund, Susan K. Wiser, Florian Wittmann, Hannsjoerg Woell, Verginia Wortel, Roderick Zagt, Tomasz Zawiła-Niedźwiecki, Chunyu Zhang, Xiuhai Zhao, Mo Zhou, Zhi-Xin Zhu, Irie C. Zo-Bi, Constantin M. Zohner

**Affiliations:** 1 Institute of Integrative Biology, ETH Zurich (Swiss Federal Institute of Technology), Zurich, Switzerland; 2 Department of Biology, Washington University, Saint Louis, MO USA; 3 Department of Forestry and Natural Resources, Purdue University, West Lafayette, IN USA; 4 Department of Agricultural and Forest Sciences and Engineering, University of Lleida, Lleida, Spain; 5 Joint Research Unit CTFC - AGROTECNIO - CERCA, Solsona, Spain; 6 Wageningen University and Research, Wageningen, the Netherlands; 7 Department of Forest Resources, University of Minnesota, St. Paul, MN USA; 8 Hawkesbury Institute for the Environment, Western Sydney University, Penrith, New South Wales Australia; 9 Institute for Global Change Biology, and School for Environment and Sustainability, University of Michigan, Ann Arbor, MI USA; 10 Chair of Crop Science and Plant Biology, Estonian University of Life Sciences, Tartu, Estonia; 11 Swiss Federal Institute for Forest, Snow and Landscape Research WSL, Birmensdorf, Switzerland; 12 UFR Biosciences, University Félix Houphouët-Boigny, Abidjan, Côte d’Ivoire; 13 Department of Agricultural, Food, Environmental and Animal Sciences, University of Udine, Udine, Italy; 14 National Biodiversity Future Center (NBFC), Palermo, Italy; 15 Spatial Ecology and Conservation Laboratory, Department of Tourism, Recreation and Sport Management, University of Florida, Gainesville, FL USA; 16 Forestry School, Tecnológico de Costa Rica TEC, Cartago, Costa Rica; 17 Fundacion ConVida, Universidad Nacional Abierta y a Distancia, UNAD, Medellin, Colombia; 18 Negaunee Integrative Research Center, Field Museum of Natural History, Chicago, IL USA; 19 Center for Tropical Research, Institute of the Environment and Sustainability, UCLA, Los Angeles, CA USA; 20 Silviculture and Forest Ecology of the Temperate Zones, University of Göttingen, Göttingen, Germany; 21 Division of Forest and Forest Resources, Norwegian Institute of Bioeconomy Research (NIBIO), Ås, Norway; 22 Museo de Historia natural Noel kempff Mercado, Santa Cruz, Bolivia; 23 European Commission, Joint Research Center, Ispra, Italy; 24 UNELLEZ-Guanare, Programa de Ciencias del Agro y el Mar, Herbario Universitario (PORT), Portuguesa, Venezuela; 25 Compensation International S. A. Ci Progress-GreenLife, Bogotá, D.C. Colombia; 26 School of Geography, University of Leeds, Leeds, UK; 27 Department of Geomatics, Forest Research Institute, Raszyn, Poland; 28 Naturalis Biodiversity Center, Leiden, the Netherlands; 29 Centro Multidisciplinar, Universidade Federal do Acre, Rio Branco, Brazil; 30 Proceedings of the National Academy of Sciences, Washington, DC USA; 31 Department of Evolutionary Anthropology, Duke University, Durham, NC USA; 32 TERRA Teach and Research Centre, Gembloux Agro Bio-Tech, University of Liege, Liege, Belgium; 33 Forestry Consultant, Grosseto, Italy; 34 Institut Agronomique néo-Calédonien (IAC), Nouméa, New Caledonia; 35 AMAP, Univ. Montpellier, Montpellier, France; 36 CIRAD, CNRS, INRAE, IRD, Montpellier, France; 37 Institute of Tropical Forest Conservation, Mbarara University of Sciences and Technology, Mbarara, Uganda; 38 Isotope Bioscience Laboratory - ISOFYS, Ghent University, Ghent, Belgium; 39 Ștefan cel Mare University of Suceava, Suceava, Romania; 40 Department of Forest Sciences, Luiz de Queiroz College of Agriculture, University of São Paulo, Piracicaba, Brazil; 41 Bavarian State Institute of Forestry, Freising, Germany; 42 Department of Natural Sciences, Manchester Metropolitan University, Manchester, UK; 43 Institute of Biology, Geobotany and Botanical Garden, Martin Luther University Halle-Wittenberg, Halle-Wittenberg, Germany; 44 German Centre for Integrative Biodiversity Research (iDiv) Halle-Jena-Leipzig, Leipzig, Germany; 45 Department of Agriculture, Food, Environment and Forest (DAGRI), University of Firenze, Florence, Italy; 46 Department of Biological, Geological, and Environmental Sciences, University of Bologna, Bologna, Italy; 47 Department of Spatial Regulation GIS and Forest Policy, Institute of Forestry, Belgrade, Serbia; 48 Department of Ecology and Evolutionary Biology, University of Connecticut, Storrs, CT USA; 49 Tropical Forest and People Research Centre, University of the Sunshine Coast, Sippy Downs, Queensland Australia; 50 Faculty of Natural Resources Management, Lakehead University, Thunder Bay, Ontario Canada; 51 Division of Forest Resources Information, Korea Forest Promotion Institute, Seoul, South Korea; 52 IFER - Institute of Forest Ecosystem Research, Jilove u Prahy, Czech Republic; 53 Global Change Research Institute CAS, Brno, Czech Republic; 54 Nicholas School of the Environment, Duke University, Durham, NC USA; 55 Department of Biology, University of Missouri-St Louis, St. Louis, MO USA; 56 Programa de Pós-graduação em Biologia Vegetal, Instituto de Biologia, Universidade Estadual de Campinas, Campinas, Brazil; 57 Department of Plant Sciences and Conservation Research Institute, University of Cambridge, Cambridge, UK; 58 Andes to Amazon Biodiversity Program, Madre de Dios, Peru; 59 Facultad de Ciencias Forestales y Ambientales, Universidad Juárez del Estado de Durango, Durango, Mexico; 60 Department of Biology, West Virginia University, Morgantown, WV USA; 61 Department of Physical and Biological Sciences, The College of Saint Rose, Albany, NY USA; 62 Biology Department, Centre for Structural and Functional Genomics, Concordia University, Montreal, Quebec Canada; 63 Natural Science Department, Universidade Regional de Blumenau, Blumenau, Brazil; 64 Cirad, UMR EcoFoG (AgroParisTech, CNRS, INRAE, Université des Antilles Université de la Guyane), Campus Agronomique, Kourou, French Guiana; 65 Department of Geography, Environment and Geomatics, University of Guelph, Guelph, Ontario Canada; 66 Institute of Forestry, Belgrade, Serbia; 67 Institute of Botany, The Czech Academy of Sciences, Třeboň, Czech Republic; 68 Department of Botany, Faculty of Science, University of South Bohemia, České Budějovice, Czech Republic; 69 IRET, Herbier National du Gabon (CENAREST), Libreville, Gabon; 70 Department of Ecology and Evolutionary Biology, University of Arizona, Tucson, AZ USA; 71 The Santa Fe Institute, Santa Fe, NM USA; 72 Queensland Herbarium and Biodiversity Science, Department of Environment and Science, Toowong, Queensland Australia; 73 Ecole de Foresterie et Ingénierie du Bois, Université Nationale d’Agriculture, Kétou, Benin; 74 School of Biological and Behavioural Sciences, Queen Mary University of London, London, UK; 75 Biology Centre of the Czech Academy of Sciences, Institute of Entomology, Ceske Budejovice, Czech Republic; 76 Geography, College of Life and Environmental Sciences, University of Exeter, Exeter, UK; 77 Museu Paraense Emílio Goeldi. Coordenação de Ciências da Terra e Ecologia, Belém, Pará Brasil; 78 Natural Resources Institute Finland (Luke), Joensuu, Finland; 79 Institute of Plant Sciences, University of Bern, Bern, Switzerland; 80 Forest Research Institute Malaysia, Kuala Lumpur, Malaysia; 81 Department of Forest Resource Management, Swedish University of Agricultural Sciences SLU, Umea, Sweden; 82 Research and Innovation Center, Fondazione Edmund Mach, San Michele All’adige, Italy; 83 Forestry Division, Food and Agriculture Organization of the United Nations, Rome, Italy; 84 Glick Designs LLC, Hadley, MA USA; 85 Royal Botanic Garden Edinburgh, Edinburgh, UK; 86 Department of Biology, University of Oxford, Oxford, UK; 87 Department of Plant Systematics, University of Bayreuth, Bayreuth, Germany; 88 Cirad, UPR Forêts et Sociétés, University of Montpellier, Montpellier, France; 89 Department of Forestry and Environment, National Polytechnic Institute (INP-HB), Yamoussoukro, Côte d’Ivoire; 90 Forest Research Institute, University of the Sunshine Coast, Sippy Downs, Queensland Australia; 91 Helmholtz GFZ German Research Centre for Geosciences, Remote Sensing and Geoinformatics Section, Telegrafenberg, Potsdam, Germany; 92 Centre for Conservation Science, The Royal Society for the Protection of Birds, Sandy, UK; 93 Wild Chimpanzee Foundation, Liberia Office, Monrovia, Liberia; 94 Instituto de Investigaciones de la Amazonía Peruana, Iquitos, Peru; 95 Centre for Invasion Biology, Department of Mathematical Sciences, Stellenbosch University, Stellenbosch, South Africa; 96 Theoretical Ecology Unit, African Institute for Mathematical Sciences, Cape Town, South Africa; 97 AMAP, Univ Montpellier, CIRAD, CNRS, INRAE, IRD, Montpellier, France; 98 National Institute of Amazonian Research, Manaus, Brazil; 99 Department of Forest Science, Tokyo University of Agriculture, Tokyo, Japan; 100 Institute of Dendrology, Polish Academy of Sciences, Kórnik, Poland; 101 Department of Game Management and Forest Protection, Poznań University of Life Sciences, Poznań, Poland; 102 Faculty of Biology, Białowieża Geobotanical Station, University of Warsaw, Białowieża, Poland; 103 Department of Geosciences and Natural Resource Management, University of Copenhagen, Copenhagen, Denmark; 104 Department of Plant Biology, Institute of Biology, University of Campinas, UNICAMP, Campinas, Brazil; 105 School of Biological Sciences, University of Bristol, Bristol, UK; 106 Forestry Faculty, Mytischi Branch of Bauman Moscow State Technical University, Mytischi, Russian Federation; 107 CAVElab-Computational and Applied Vegetation Ecology, Department of Environment, Ghent University, Ghent, Belgium; 108 CTFS-ForestGEO, Smithsonian Tropical Research Institute, Balboa, Panama; 109 Department of Physical and Environmental Sciences, Colorado Mesa University, Grand Junction, CO USA; 110 UniSA STEM and Future Industries Institute, University of South Australia, Adelaide, South Australia Australia; 111 Department of Botany, Dr Harisingh Gour Vishwavidyalaya (A Central University), Sagar, India; 112 Department of Agriculture, Forestry and Bioresources, Seoul National University, Seoul, South Korea; 113 Interdisciplinary Program in Agricultural and Forest Meteorology, Seoul National University, Seoul, South Korea; 114 National Center for Agro Meteorology, Seoul, South Korea; 115 Research Institute for Agriculture and Life Sciences, Seoul National University, Seoul, South Korea; 116 Graduate School of Agriculture, Kyoto University, Kyoto, Japan; 117 Institute for World Forestry, University of Hamburg, Hamburg, Germany; 118 Institute of Forestry and Engineering, Estonian University of Life Sciences, Tartu, Estonia; 119 Biodiversity and Natural Resources Program, International Institute for Applied Systems Analysis, Laxenburg, Austria; 120 Peoples Friendship University of Russia (RUDN University), Moscow, Russian Federation; 121 Department of Geography, University College London, London, UK; 122 Faculty of Forestry, Qingdao Agricultural University, Qingdao, China; 123 Center for Forest Ecology and Productivity, Russian Academy of Sciences, Moscow, Russian Federation; 124 Environmental Change Institute, School of Geography and the Environment, University of Oxford, Oxford, UK; 125 AgroParisTech, UMR-AMAP, Cirad, CNRS, INRA, IRD, Université de Montpellier, Montpellier, France; 126 Departamento de Ciências Biológicas, Universidade do Estado de Mato Grosso, Nova Xavantina, Brazil; 127 Department of Environment and Geography, University of York, York, UK; 128 Flamingo Land Ltd, Kirby Misperton, UK; 129 Department of Wildlife Management, College of African Wildlife Management, Mweka, Tanzania; 130 Departamento de Ecología y Recursos Naturales, Facultad de Ciencias, Universidad Nacional Autónoma de México, Mexico City, Mexico; 131 Universidad del Tolima, Ibagué, Colombia; 132 Colegio de Profesionales Forestales de Cochabamba, Cochabamba, Bolivia; 133 Jardín Botánico de Missouri, Pasco, Peru; 134 Universidad Nacional de San Antonio Abad del Cusco, Cusco, Peru; 135 Department of Environment and Development Studies, United International University, Dhaka, Bangladesh; 136 Instituto de Silvicultura e Industria de la Madera, Universidad Juárez del Estado de Durango, Durango, Mexico; 137 Programa de doctorado en Ingeniería para el desarrollo rural y civil, Escuela de Doctorado Internacional de la Universidad de Santiago de Compostela (EDIUS), Santiago de Compostela, Spain; 138 Universidad Estatal Amazónica, Puyo, Pastaza Ecuador; 139 Department of Evolutionary Biology and Environmental Studies, University of Zürich, Zurich, Switzerland; 140 Climate, Fire, and Carbon Cycle Sciences, USDA Forest Service, Durham, NC USA; 141 Centre for Forest Research, Université du Québec à Montréal, Montréal, Québec Canada; 142 V. N. Sukachev Institute of Forest, FRC KSC, Siberian Branch of the Russian Academy of Sciences, Krasnoyarsk, Russian Federation; 143 Forest Ecology and Forest Management Group, Wageningen University and Research, Wageningen, the Netherlands; 144 Department of Ecology and Environmental Sciences, Pondicherry University, Puducherry, India; 145 Instituto Nacional de Tecnología Agropecuaria (INTA), Universidad Nacional de la Patagonia Austral (UNPA), Consejo Nacional de Investigaciones Científicas y Técnicas (CONICET), Río Gallegos, Argentina; 146 School of Social Sciences (Urban Studies), Western Sydney University, Penrith, New South Wales Australia; 147 GIP Ecofor, Paris, France; 148 Instituto Nacional de Pesquisas da Amazônia, Manaus, Brazil; 149 Laboratório de Dendrologia e Silvicultura Tropical, Centro de Formação em Ciências Agroflorestais, Universidade Federal do Sul da Bahia, Itabuna, Brazil; 150 Field Museum of Natural History, Chicago, IL USA; 151 Jardín Botánico de Medellín, Medellin, Colombia; 152 The Nature Conservancy, Boulder, CO USA; 153 Chair for Forest Growth and Yield Science, Department of Life Science Systems, TUM School for Life Sciences, Technical University of Munich, Freising, Germany; 154 Sustainable Forest Management Research Institute iuFOR, University Valladolid, Valladolid, Spain; 155 Universidad Nacional de la Amazonía Peruana, Iquitos, Peru; 156 Servicios Ecosistémicos y Cambio Climático (SECC), Fundación Con Vida and Corporación COL-TREE, Medellín, Colombia; 157 Centro Agricoltura, Alimenti, Ambiente, University of Trento, San Michele All’adige, Italy; 158 Center for Natural Climate Solutions, Conservation International, Arlington, VA USA; 159 Department of Biology, University of Florence, Florence, Italy; 160 Tropical Biodiversity, MUSE - Museo delle Scienze, Trento, Italy; 161 Info Flora, Geneva, Switzerland; 162 Department of Environmental Sciences, Central University of Jharkhand, Ranchi, Jharkhand India; 163 Centro de Modelación y Monitoreo de Ecosistemas, Universidad Mayor, Santiago, Chile; 164 Vicerrectoría de Investigación y Postgrado, Universidad de La Frontera, Temuco, Chile; 165 Departamento de Silvicultura y Conservación de la Naturaleza, Universidad de Chile, Temuco, Chile; 166 Rhino and Forest Fund e.V., Kehl, Germany; 167 Siberian Federal University, Krasnoyarsk, Russian Federation; 168 Geobotany, Faculty of Biology, University of Freiburg, Freiburg im Breisgau, Germany; 169 Department of Geography, Remote Sensing Laboratories, University of Zürich, Zurich, Switzerland; 170 National Forest Centre, Forest Research Institute Zvolen, Zvolen, Slovakia; 171 Université de Lorraine, AgroParisTech, INRAE, Silva, Nancy, France; 172 Center for Biodiversity Dynamics in a Changing World (BIOCHANGE), Department of Biology, Aarhus University, Aarhus, Denmark; 173 Faculty of Environmental Sciences and Natural Resource Management, Norwegian University of Life Sciences, Ås, Norway; 174 Departamento de Biología, Universidad de la Serena, La Serena, Chile; 175 Centro de Ciências Biológicas e da Natureza, Universidade Federal do Acre, Rio Branco, Acre Brazil; 176 Guyana Forestry Commission, Georgetown, French Guiana; 177 Environmental and Life Sciences, Faculty of Science, Universiti Brunei Darussalam, Gadong, Brunei Darussalam; 178 Plant Systematic and Ecology Laboratory, Department of Biology, Higher Teachers’ Training College, University of Yaoundé I, Yaoundé, Cameroon; 179 Departamento de Ecologia, Universidade Federal do Rio Grande do Norte, Natal, Rio Grande do Norte Brazil; 180 Department of Forest Management, Dendrometry and Forest Economics, Warsaw University of Life Sciences, Warsaw, Poland; 181 Center for Ecological Dynamics in a Novel Biosphere (ECONOVO) & Center for Biodiversity Dynamics in a Changing World (BIOCHANGE), Department of Biology, Aarhus University, Aarhus C, Denmark; 182 Section for Ecoinformatics and Biodiversity, Department of Biology, Aarhus University, Aarhus, Denmark; 183 Faculty of Forestry and Wood Sciences, Czech University of Life Sciences, Prague, Czech Republic; 184 Wildlife Conservation Society, New York, NY USA; 185 Quantitative Biodiversity Dynamics, Department of Biology, Utrecht University, Utrecht, the Netherlands; 186 Iwokrama International Centre for Rainforest Conservation and Development (IIC), Georgetown, French Guiana; 187 School of Forestry and Environmental Studies, Yale University, New Haven, CT USA; 188 Botanical Garden of Ural Branch of Russian Academy of Sciences, Ural State Forest Engineering University, Yekaterinburg, Russian Federation; 189 Pontificia Universidad Católica del Ecuador, Quito, Ecuador; 190 LINCGlobal, Museo Nacional de Ciencias Naturales, CSIC, Madrid, Spain; 191 Plant Ecology and Nature Conservation Group, Wageningen University, Wageningen, the Netherlands; 192 Silviculture Research Institute, Vietnamese Academy of Forest Sciences, Hanoi, Vietnam; 193 Department of Biology, Stanford University, Stanford, CA USA; 194 Agricultural High School, ESAV, Polytechnic Institute of Viseu, IPV, Viseu, Portugal; 195 Centre for the Research and Technology of Agro-Environmental and Biological Sciences, CITAB, UTAD, Quinta de Prados, Vila Real, Portugal; 196 Department of Forest Engineering, Universidade Regional de Blumenau, Blumenau, Brazil; 197 Environmental Studies and Research Center, University of Campinas, UNICAMP, Campinas, Brazil; 198 Department of Forest and Wood Science, University of Stellenbosch, Stellenbosch, South Africa; 199 Key Laboratory of Tropical Biological Resources, Ministry of Education, School of Life and Pharmaceutical Sciences, Hainan University, Haikou, China; 200 Division of Forestry and Natural Resources, West Virginia University, Morgantown, WV USA; 201 Department of Zoology, University of Oxford, Oxford, UK; 202 Manaaki Whenua–Landcare Research, Lincoln, New Zealand; 203 Department of Wetland Ecology, Institute for Geography and Geoecology, Karlsruhe Institute for Technology, Karlsruhe, Germany; 204 Independent Researcher, Sommersbergseestrasse, Bad Aussee, Austria; 205 Centre for Agricultural Research in Suriname (CELOS), Paramaribo, Suriname; 206 Tropenbos International, Wageningen, the Netherlands; 207 Polish State Forests, Coordination Center for Environmental Projects, Warsaw, Poland; 208 Research Center of Forest Management Engineering of State Forestry and Grassland Administration, Beijing Forestry University, Beijing, China; 209 Present Address: Department of Genetics, Evolution, and Environment, University College London, London, United Kingdom

**Keywords:** Ecology, Biogeography

## Abstract

Understanding what controls global leaf type variation in trees is crucial for comprehending their role in terrestrial ecosystems, including carbon, water and nutrient dynamics. Yet our understanding of the factors influencing forest leaf types remains incomplete, leaving us uncertain about the global proportions of needle-leaved, broadleaved, evergreen and deciduous trees. To address these gaps, we conducted a global, ground-sourced assessment of forest leaf-type variation by integrating forest inventory data with comprehensive leaf form (broadleaf vs needle-leaf) and habit (evergreen vs deciduous) records. We found that global variation in leaf habit is primarily driven by isothermality and soil characteristics, while leaf form is predominantly driven by temperature. Given these relationships, we estimate that 38% of global tree individuals are needle-leaved evergreen, 29% are broadleaved evergreen, 27% are broadleaved deciduous and 5% are needle-leaved deciduous. The aboveground biomass distribution among these tree types is approximately 21% (126.4 Gt), 54% (335.7 Gt), 22% (136.2 Gt) and 3% (18.7 Gt), respectively. We further project that, depending on future emissions pathways, 17–34% of forested areas will experience climate conditions by the end of the century that currently support a different forest type, highlighting the intensification of climatic stress on existing forests. By quantifying the distribution of tree leaf types and their corresponding biomass, and identifying regions where climate change will exert greatest pressure on current leaf types, our results can help improve predictions of future terrestrial ecosystem functioning and carbon cycling.

## Abstract (web summary)

Integrating inventory data with machine learning models reveals the global composition of tree types—needle-leaved evergreen individuals dominate, followed by broadleaved evergreen and deciduous trees—and climate change risks.

## Main

Forest ecosystems, which contain 80–90% of global terrestrial plant biomass^1,2^ and a large proportion of terrestrial biodiversity^3^, regulate global biogeochemical cycles, and provide critical ecosystem services^4^. Leaves mediate forest energy and carbon inputs via photosynthesis, respiration, transpiration^5,6^ and litterfall^7,8^, thereby regulating ecosystem structure and function, and water, nutrient and carbon cycles^9–11^. Leaves of trees are highly diverse but can be broadly classified into four major types on the basis of leaf habit (evergreen vs deciduous) and form (broadleaved vs needle-leaved). These characteristics are linked to a vast array of functional traits associated with resource-use strategies and strongly depend on local growing conditions^12–15^. Therefore, understanding variation in leaf types along environmental gradients is critical to predicting global biogeochemical cycles and ecosystem functioning in a changing world. Yet, we still lack a global, quantitative understanding of forest leaf habit and form, informed by field-based observations.

Deciduous tree species evolved to tolerate seasonal climates and maximize the use of a short growing season^16,17^. They usually have higher photosynthetic rates^18^ than evergreen species and reduce transpiratory water loss due to respiration by shedding their leaves during unfavourable seasons^11^. Evergreen trees with longer leaf lifespans, by contrast, tend to have greater leaf construction costs^19^ and lower nutrient cycling rates^20^. Growing season water-use strategy commonly differs between broadleaved and needle-leaved species^21^, with needle-leaved species often showing conservative strategies^22^, such as lower stomatal conductance^23^ and higher hydraulic safety margins^24^, resulting in low photosynthesis rates^25^. A spatially explicit understanding of tree leaf types is therefore critical for estimating the sensitivity and resilience of forests to future climate and soil conditions^26–28^, and understanding the ecological consequences of such changes.

Theoretical models^29^ and remote sensing observations^30,31^ have shown general trends in how climate and soil conditions affect the geographic occurrence of broadleaved and needle-leaved tree species at regional and global scales^32^. These relationships form the foundation of dynamic global vegetation models^31,33–37^. Yet, the relative importance of various environmental characteristics on leaf habit and form remains to be determined. Furthermore, until now, these vegetation models have lacked the ground data needed to build tree-density-based ‘bottom-up’ models. Such models are crucial for validating satellite-derived trends on a global scale and for providing a comprehensive, high-resolution depiction of forest leaf-type variation across environmental gradients.

**Fig. 1:**
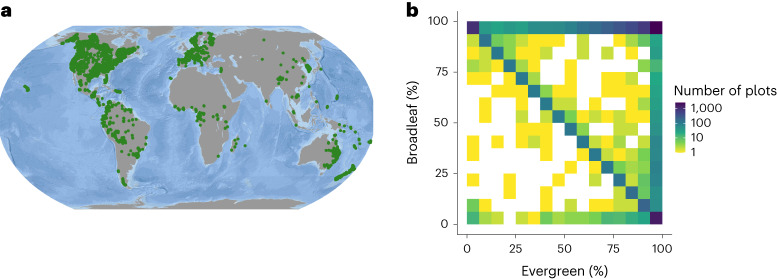
Global coverage of forest inventory locations (GFBi data) and plot-level leaf-type proportions. **a**, A total of 9,781 forest inventory plots (green points) were used for geospatial modelling of forest leaf types. **b**, Number of plots in relation to their proportion of evergreen vs deciduous and broadleaved vs needle-leaved individuals.

Here we analyse the global distribution of needle-leaved, broadleaved, evergreen and deciduous tree species, by integrating ground-sourced information from 9,781 standardized forest inventory plots in the Global Forest Biodiversity initiative (GFBi)^38^ database with species-level leaf habit (evergreen vs deciduous) and leaf form (broadleaf vs needle-leaf) information accessed from the TRY plant trait database^39^ (Fig. 1a). The 9,781 forest inventory plots represented a subsample of the full GFBi data of >1 million records to ensure an equal representation of different forest biomes across the globe and avoid modelling bias caused by uneven spatial sampling (see Methods). Using information on both the occurrences of individual trees per plot and the basal-area weighted occurrences of each individual, we calculated plot-level leaf-type proportions (1) on the basis of the leaf type of each individual tree (tree-based leaf type) and (2) by weighting each tree by its basal area (area-based leaf type) (see Methods section 'Tree leaf-type data'). The first estimate allowed us to map the tree densities of each leaf type, while the second estimate allowed us to map the leaf types represented by the largest trees in a plot. To interpolate patterns across the globe, we combined our plot-level forest leaf information with 58 environmental variables, representing vegetation characteristics, climate, topography, vegetation, soil conditions and human-related features. We also tested the relative importance of 29 commonly studied variables on leaf-type variation and characterized the relationships between environmental features and leaf type.

## Mapping global forest leaf types

To characterize the variation in forest leaf type, we first summarized the proportion of evergreen vs deciduous (leaf habit) and broadleaved vs needle-leaved (leaf form) individuals within each plot (Fig. 1a). Across all 9,781 forest plots, 13% contain exclusively broadleaved evergreen trees, 7% contain exclusively broadleaved deciduous trees, 12% contain exclusively needle-leaved evergreen trees, and the remaining 68% contain a mixture of leaf habits and forms (Fig. 1b).

**Fig. 2:**
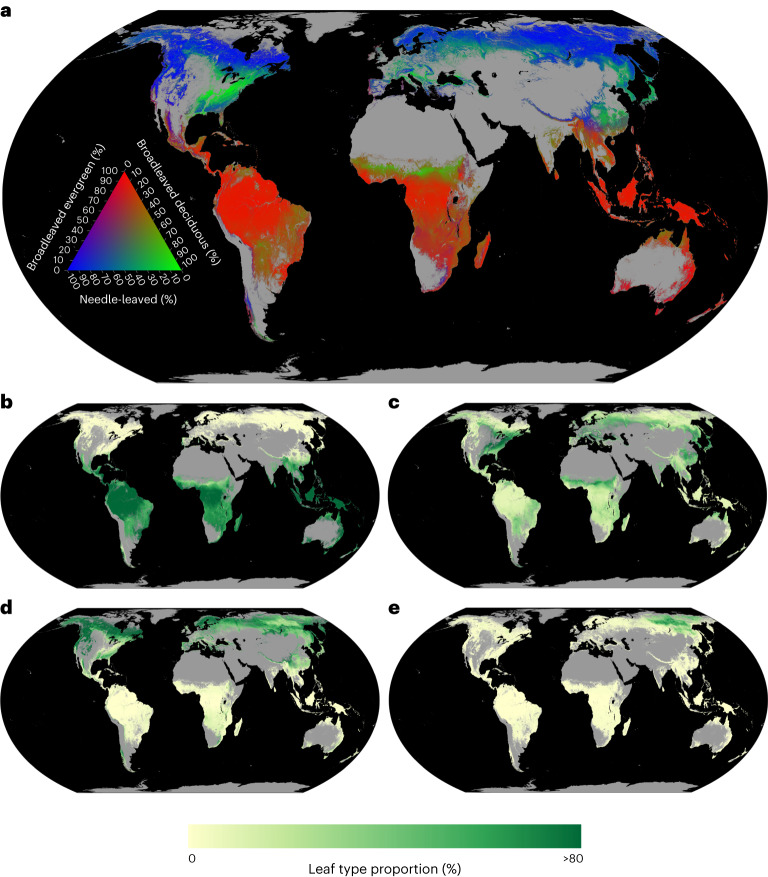
The global distribution of forest leaf types. **a**, The global distribution of tree leaf type as predicted by a random forest model built from area-based leaf-type data (see Methods). Pixels are coloured in the red, green and blue spectrum according to the percentage of total tree basal area occupied by broadleaved evergreen, broadleaved deciduous and needle-leaved tree types, as indicated by the ternary plot. Needle-leaved evergreen and needle-leaved deciduous forests were combined due to the low global coverage of needle-leaved deciduous trees. **b**–**e**, Predicted relative coverage of each leaf type from random forest models. Ref. ^81^ was used to mask non-forest areas. **b**, Broadleaved evergreen coverage. **c**, Broadleaved deciduous coverage. **d**, Needle-leaved evergreen coverage. **e**, Needle-leaved deciduous coverage.

Our random forest model predicted 75% of the global variation of forest leaf-type classes (10-fold cross-validation ${R}_{{\rm{BC}}}^{2}$, see Methods). We also ran spatially buffered leave-one-out cross-validation (LOO-CV) to account for the potential effect of spatial autocorrelation on model evaluation statistics, which resulted in a coefficient of determination (*R*^2^) of 0.56 at a buffer radius of 300 km (see Methods and Supplementary Fig. 1). Within each class, our model explained 90%, 59%, 75% and 29% (10-fold cross-validation *R*^2^) of the global variation in the proportion of broadleaved evergreen, broadleaved deciduous, needle-leaved evergreen and needle-leaved deciduous trees within forests, respectively. These predictive relationships were then used to upscale the observations across the global extent of forest coverage (Fig. 2).

To evaluate model robustness, we tested its performance on an independent validation dataset containing 3,895 sites across the globe^40^, resulting in an *R*^2^ of 0.47 (see Methods section 'Cross-validation using external data'). In addition, we compared our model output with annual land cover maps from the European Space Agency’s Climate Change Initiative (ESA CCI LC)^41^. Across all leaf types, our model showed high correlations with the ESA CCI LC outputs, with an ${R}_{{\rm{BC}}}^{2}$ of 0.61. Within each leaf type class, our model explained 78% of the variation in the proportion of broadleaved evergreen trees, 31% of broadleaved deciduous tree proportions, 64% of needle-leaved evergreen tree proportions and 19% of needle-leaved deciduous tree proportions (*R*^2^).

While considerable uncertainties exist for individual predictions at the pixel level, these uncertainties rapidly decrease as the model is projected to a larger area (<5% at 250 km^2^; Supplementary Fig. 2). Our model shows high confidence in tropical and boreal forests, whereas predictive confidence is lower in mixed forests and ecotones between different types of forest (Supplementary Figs. 3 and 4). For example, models for broadleaved evergreen and deciduous species share low predictive confidence in central African savanna regions. Similarly, low predictive confidence can be found across eastern Russian mixed forests. The low predictive confidence for these regions can be attributed to low sample size and mixed occurrence of broadleaved evergreen and deciduous species, as well as differences in the year of observation across forest survey plots, which may lead to larger uncertainties in ecotones where forest types can shift in relatively short time periods.

## Global environmental drivers of forest leaf-type variation

**Fig. 3:**
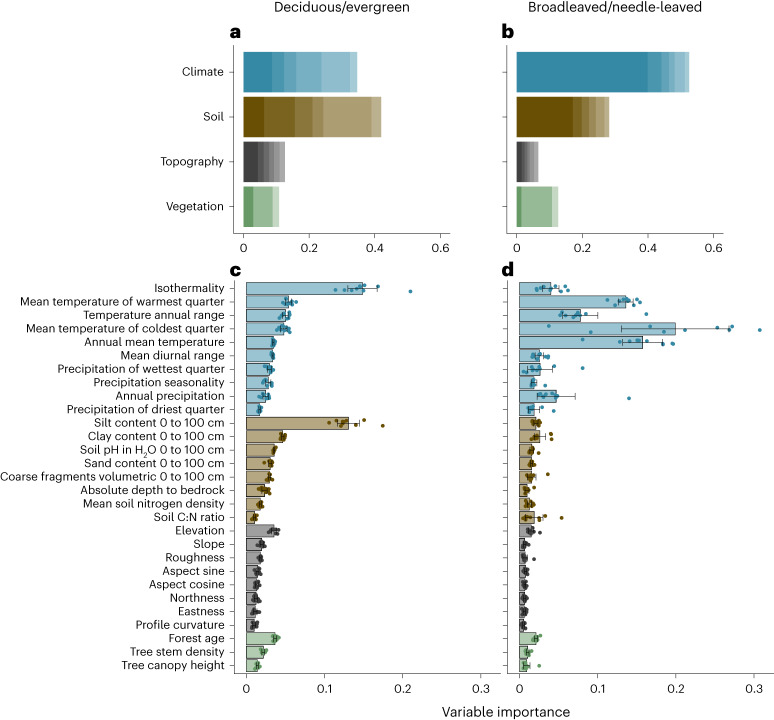
Variable importance of environmental covariates on forest leaf-type variation. **a**,**b**, Cumulative importance of the first six principal components of climate, soil, topographic and vegetation covariates in the variation of leaf habit (**a**) and leaf form (**b**). **c**,**d**, Variable importance of selected environmental features on variation in leaf habit (**c**) and leaf form **(d**). Bars in **c** and **d** represent the mean ± 95% CI; relative importance based on the 10 best random forest models (*n* = 10; see Methods). Area-based leaf-type proportions were used to represent forest (plot-level) leaf-type variation.

To assess the relative importance of environmental features on variation in leaf types, we ran random forest models including a range of environmental variables (see Methods). We combined these environmental factors into three groups (climate, soil and topography). To test for the relative importance of climate, soil and topographic characteristics, we ran a principal component analysis (PCA) for each of these variable groups and selected the first six principal components, which explained ≥90% of the total variation across all variables. Our analyses show that climate and soil characteristics jointly determine the global leaf-type distribution (Fig. 3a). With respect to variation in leaf habit, temperature fluctuations, that is, isothermality and temperature seasonality, were the most important variables (Fig. 3c). Yet, the entire combination of soil features (first six principal components of soil variables) was as important as climate for predicting leaf habit in our random forest model (Fig. 3a), suggesting that soil characteristics play an important role in the global distribution of evergreen ‘vs’ deciduous species. Especially soil texture, in combination with pH, appears to affect global variation in tree leaf habit. Acidic soils, commonly found in tropical and boreal regions, inhibit nutrient (N and P) supply by reducing cation availability and limiting tree growth rates^16^. This might explain why broadleaved deciduous species that require high nutrient supply are less abundant in regions with acidic soil. Broadleaved evergreen species, by contrast, may better cope with nutrient poor, acidic soils^42^. Similarly, needle-leaved evergreen species that can maintain growth even under low nutrient supply are more abundant in regions with acidic soil^16^. The high tannin and phenol contents of needle leaves further contribute to the acidification of soils^16^, probably creating a positive feedback towards coniferous dominance. Overall, our results point towards a feedback between tree leaf habits and soil conditions, highlighting the connection between physical soil features and soil water^43^ and nutrient^44^ availability.

Variation in leaf form was best predicted by climate variables (Fig. 3b), with the most important variable being temperature of the coldest quarter (Fig. 3d). By contrast to leaf habit, soil, topographic and vegetation features were less important in driving variation in leaf form, indicating adaptation to extreme climates, cold winters or extended dry seasons, as a major determinant of leaf form. This supports previous studies indicating that diverse leaf forms evolved to adapt to different climates^45,46^.

## Computation of tree densities with different leaf types

**Fig. 4:**
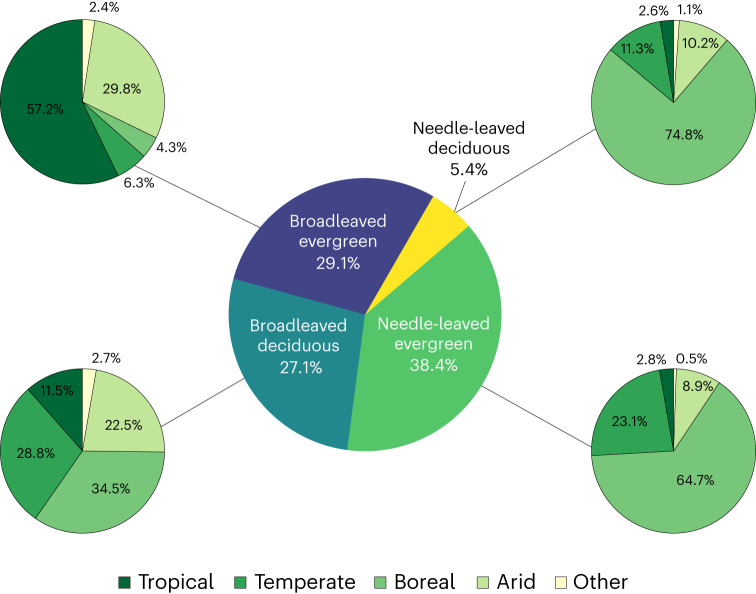
The global proportion of evergreen broadleaved, deciduous broadleaved, needle-leaved evergreen and needle-leaved deciduous trees. The relative proportions of trees that occur within tropical, temperate, boreal and arid regions are shown as separate pie charts for each leaf type.

To quantify the proportions of different leaf types across global forests, we combined a global tree density distribution map^47^ with our individual-based leaf type models (see Methods and Supplementary Fig. 5). At the global scale, we estimate that of the ∼3 trillion adult trees presently existing, 29.1% (95% CI = 27.5–30.7%) are broadleaved evergreen, 27.1% (23.8–30.6%) are broadleaved deciduous, 38.4% (35.2–41.6%) are needle-leaved evergreen and 5.4% (4.3–6.6%) are needle-leaved deciduous (Fig. 4 and Supplementary Fig. 6). Even though needle-leaved tree species comprise less than 2% of the world’s estimated 73,000 tree species^48^, this small fraction of diversity represents around 44% of individual trees on Earth.

Of the 1.15 trillion needle-leaved evergreen trees growing worldwide, the majority (64.7%) are found in boreal regions, while 23.1%, 8.9%, 2.8% are found in temperate, arid and tropical regions, respectively (Fig. 4). In contrast, of the 0.87 trillion broadleaved evergreen trees growing worldwide, the majority exists in tropical (57.2%) and arid (29.8%) regions, with 6.3% and 4.3% existing in temperate and boreal regions, respectively (Fig. 4). Broadleaved deciduous trees show the widest range of occurrences. Of the 0.81 trillion broadleaved deciduous trees, 34.5% are found in boreal regions, 28.8% in temperate regions, 22.5% in arid regions and 11.5% in tropical regions (Fig. 4). We further estimate that there are 0.16 trillion needle-leaved deciduous trees across the globe, the vast majority of which grow in boreal regions.

Using our basal-area-based model of leaf types, we were able to estimate the biomass contribution of each leaf type within individual forest pixels by integrating our data with a recently published aboveground biomass map^49^. Our analysis revealed that broadleaved evergreen trees store the largest proportion of global forest biomass, accounting for 54.4% (335.7 Gt) out of the total biomass of 617 Gt. Broadleaved deciduous trees contribute 22.1% (136.2 Gt), needle-leaved evergreen trees contribute 20.5% (126.4 Gt) and needle-leaved deciduous trees contribute 4% (18.7 Gt) (Supplementary Fig. 7). Interestingly, despite there being 42% more evergreen needle-leaved trees compared with broadleaved evergreen trees, their biomass contribution is 62% (209.3 Gt) lower. This distribution of biomass across different leaf types provides valuable insights into the carbon storage capacity of diverse forest ecosystems.

## Climatic risk assessment of future leaf types

Climate change will strongly affect the functioning of terrestrial ecosystems by altering growth, mortality and reproduction of trees and their interactions with leaf form and habit^16^. Our models allowed us to highlight areas of potential risk by identifying the regions where future climates will shift to conditions that currently support leaf types different from the prevailing ones. In these regions, trees are likely to experience more climatic stress in the future. To assess the extent and distribution of future changes in forest leaf-type climate envelopes, we projected our leaf-type models into the future using three climate change scenarios (low-emission scenario (SSP1–RCP2.6), business-as-usual scenario (SSP3–RCP7) and high-emission scenario (SSP5–RCP8.5))^50^. To do so, we used our random forest models of present leaf type distributions and replaced all climate variables reflecting the 1979–2013 climate (see Supplementary Fig. 1) with climate model projections for 2071–2100 while keeping soil, topographic, vegetative and anthropogenic characteristics constant.

**Fig. 5:**
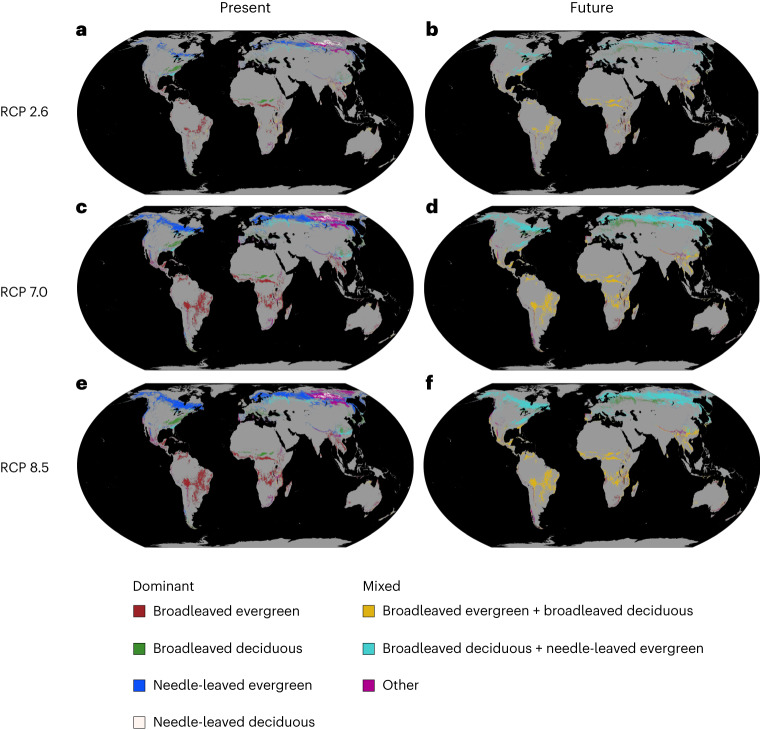
Forested areas where future climates may no longer support prevailing leaf types. If a pixel’s forest area was predominantly (>60%) covered by one leaf type, it was classified as that specific leaf type. Pixels where no leaf type exceeded 60% coverage were classified as mixed forest. To determine the relative proportion of each leaf type per plot, we considered the basal area of individual trees (area-based leaf type). Coloured pixels on the map indicate areas that, by the end of the century (2071–2100), will face climate conditions that currently support a different forest type. The future climate conditions were represented using three climate change scenarios: low-emission (SSP1–RCP2.6; **a**,**b**), business-as-usual (SSP3–RCP7; **c**,**d**) and high-emission (SSP5–RCP8.5; **e**,**f**) for the period 2071–2100. Panels **a**, **c** and **e** show the present forest types. In contrast, panels **b**, **d** and **f** show the type of forest expected under the projected future climate of each respective pixel.

The results suggest that forests will experience substantial shifts in leaf-type climate conditions. Depending on future emissions pathways, 17 to 34% of future forested regions are projected to experience a climate by the end of the century that currently supports leaf types different from the prevailing ones (Fig. 5 and Supplementary Fig. 8; see Supplementary Fig. 9 for an alternative definition of forest types). The climate conditions that have historically supported evergreen forests are declining as global conditions shift towards those that have historically supported more deciduous forests, and this appears to be the case for both broadleaved and needle-leaved species (Supplementary Fig. 10). Specifically, 7–20% of the broadleaved evergreen forests are likely to experience changes towards conditions that currently support deciduous species (Fig. 5). Similarly, 29–67% of the needle-leaved evergreen forests will experience changes towards climate conditions that currently support mixed or deciduous forests (Fig. 5 and Supplementary Figs. 8–12). If these climate projections are realized, plants in those regions must either tolerate more stressful environmental conditions or shift their distributions, causing changes in forest composition^51^. Previous studies predicting ecoregion shifts have also shown a heightened vulnerability to changing climate conditions, surpassing even the susceptibility of leaf types^52^.

It is important to acknowledge that linking forest leaf types to climate alone cannot fully capture the complex interactions of other influential factors, including CO_2_ concentration^53,54^ and nutrient availability^26^. Consequently, the analysis presented in this study does not project actual changes in forest leaf types. Instead, its focus is on identifying regions where future climates will shift to conditions that currently support different leaf types than what is currently observed. Different species may exhibit varying tolerance thresholds and responses to CO_2_ fertilization, leading to divergent outcomes. Moreover, it is possible that species sharing the same leaf habit or form but with broader climatic tolerance ranges could replace the present species, potentially mitigating leaf-type changes.

Our analysis serves as a risk assessment, highlighting regions where climate poses a potential threat to the existing forest composition. Further research is necessary to gain a deeper understanding of the intricate interactions between climatic changes, elevated atmospheric CO_2_ concentrations and nutrient availability. These interactions play a pivotal role in determining essential aspects of forest ecology, such as germination rates, seedling survival, growth rates and tree mortality, which ultimately shape forest composition. Nonetheless, the analysis underscores the substantial and rapid changes in climatic conditions that forests are already experiencing and will continue to experience even more profoundly in the future, on timescales ranging from decades to centuries.

## Methodological considerations

Our models successfully captured a substantial portion of the observed spatial variation in forest leaf types, with an overall explained variation of 75%. However, the accuracy of our predictions varied across different leaf types. Among them, our model achieved the highest accuracy in predicting the spatial distribution of broadleaved evergreen tree species, with an explained variation of 90%. Conversely, our model explained only 29% of the variation in needle-leaved deciduous trees, which can be attributed to the limited availability of data from plots containing needle-leaved deciduous species.

To address the uncertainties associated with our predictions, we employed a subsampling approach, running 100 independent models. This allowed us to assess the range of variability in our predictions and identify areas with lower predictive confidence. The resulting maps of model uncertainty (Supplementary Fig. 3) highlight regions where caution should be exercised when interpreting our predictions.

While our study supports many of the mechanisms identified in previous research, our correlative analyses do not necessarily establish causal relationships. To map leaf-ype variation across the globe on the basis of the relationships with environmental features, we used global layers. This approach was necessary as point-level predictor variables cannot be used to interpolate predictions across the globe. While the majority of these layers effectively capture local variations, soil layers may not fully reflect point-level soil observations due to the inherent spatial heterogeneity of soil conditions. In addition, soil layers are typically derived from interpolation methods using environmental information and may thus be correlated with climate variables. While our random forest model predictions are not affected by multicollinearity, this could impact the quantification of variable importance related to environmental, soil, topographic and vegetation features.

Incorporating local point observations into the model training was not feasible because the ground-based forest inventory data we used did not include field-measured environmental and soil characteristics. Although we examined additional point-level observations from the World Soil Information Service (WOSIS) dataset (see next paragraph), these observations often did not align spatially with the forest inventory plots, resulting in a reduced sample size (>80% reduction) and geographic bias (Fig. 1 and Supplementary Fig. 14). Moreover, training the model using point observations while relying on soil layers for model prediction could introduce further uncertainties and biases. To avoid these limitations, we conducted both model training and predictions using the same soil layers.

Nevertheless, we conducted analyses to determine the agreement between results based on global soil layers and point-level soil observations. We matched our forest inventory dataset^55^ with the WOSIS dataset^56^, which contains local point observations of soil features. These analyses indicated that: (1) model predictions remained consistent when using point observations instead of global layers of soil characteristics (97–99% similarity in predictions), (2) the global soil layers exhibited good agreement with point observations for most soil variables, particularly the four most important variables (*R*^2^ = 0.42–0.62; Fig. 3c and Supplementary Fig. 13) and (3) the inferred importance of soil features in leaf type variation remained similar (<5% difference) when using point observations instead of soil layers as predictors (Supplementary Fig. 14). These analyses underscore the crucial role of soil features in determining global leaf-type variation.

## Conclusions

By characterizing the environmental controls of forest leaf-type variation and integrating this information with tree density data, our analysis reveals that 38% of global tree individuals are needle-leaved evergreen, 29% are broadleaved evergreen, 27% are broadleaved deciduous and 5% are needle-leaved deciduous. We further show predictable global patterns in forest leaf varieties, with the relative abundance of leaf forms mainly correlating with temperature variation. In contrast, leaf habit is jointly controlled by climate and soil characteristics. These global relationships between environmental factors and forest leaf types largely agree with local experimental and modelling studies^57–59^, which also highlight the dual role of climate and soil conditions in driving variation in forest types.

Our analysis of the spatial variation of forest leaf types refines our understanding of forest composition and structure^60,61^ at a global scale. While satellite-derived approaches have been foundational for the characterization of spatial variation in canopy structure^62^, our bottom-up model, derived from empirical inventory data, allowed us to create models of forest leaf type at the individual tree level to provide novel insights into forest composition. This can help benchmark satellite-derived models of forest structure and inform ecological models of plant productivity, biogeochemical cycling, carbon storage and species distribution^31,63,64^. By identifying the main environmental characteristics that determine habitat suitability, such as annual mean temperature, climate seasonality and water and nutrient supplies, our baseline estimates of leaf-type densities are also critical for projecting population- and community-level tree demographics under current and future climate change. Ultimately, these insights can help make informed decisions to guide global efforts to conserve, restore and sustainably manage forest ecosystems that are so vital for the wellbeing of all organisms on Earth.

## Methods

### Data collection

#### Forest inventory data

To obtain empirical information on tree occurrences, we extracted a total of 1.1 million forest inventory plots with more than 50 million individual occurrence records, covering all continents except Antarctica from the GFBi dataset^38^. To avoid modelling bias caused by uneven spatial sampling across biomes, we downsampled the dataset so that the relative proportion of plots in each biome in the dataset approximately matched the proportion of forested area within each biome^65^. This was done by retaining all tropical forest plots in GFBi (*n* = 11,367) and randomly downsampling the remaining biomes in proportion to this number. Individuals with stem diameters <10 cm were excluded as the focus was on adult trees, and only plots with ≥10 adult individuals were included in the final analysis. For each plot, the dataset contains information on the location (coordinates), the year(s) when the inventory took place, stem diameter at breast height (DBH) and species identity of each individual. For plots with time series data, only the most recent observation year was included in the analysis. The average year of observation across all plots was 2005. This resulted in a total of 9,781 plots with a median size of ∼500 m^2^ and 817,091 individual tree records, with 20.3% of the plots in the boreal biome (vs 21% of forested land globally), 20.4% in temperate biomes (vs 22%), 54% in tropical biomes (vs 50%) and 3% in Mediterranean woodland, tundra, xeric shrubland or mangrove biomes (vs 7% globally).

#### Tree leaf-type data

Information on leaf habit (evergreen vs deciduous) and leaf type (broadleaved vs needle-leaved) was extracted from the TRY plant trait database^39^. For each species and genus, we assigned the most common leaf habit across all TRY observations, treating leaf habit as a binary variable of whether it is evergreen or not. Species names were standardized using the Taxonstand R package^66^. We then assigned leaf-type information to the individuals included in the GFBi dataset using species-level information or genus-level information in case species-level information was not available. Plots in which <50% of all individuals had a leaf-type record in TRY were excluded. Out of 10,274 species recorded globally in the GFBi dataset, leaf-type records could be assigned to 8,642 tropical tree species, 453 temperate tree species, 46 boreal tree species and 1,124 tree species in dry areas. Next, we calculated the proportion of each leaf-type combination (evergreen broadleaved, deciduous broadleaved, needle-leaved evergreen or needle-leaved deciduous) within a plot, either by dividing the sum of individuals featuring the respective leaf type by the total number of individuals within the plot (individual-based leaf type) or by dividing the basal area featuring the respective leaf type by the total basal area of all trees in the plot (area-based leaf type).

#### Environmental covariates

In total, 58 global environmental layers reflecting climate^50^, topography, vegetation, anthropogenic and soil^67^ (at 0 cm to 100 cm depth) characteristics were used as covariates in our analyses (Supplementary Fig. 1). Climate variables reflect the average climate between 1979–2013. All layers were standardized to 30-arc-second resolution (∼1 km^2^ at the equator).

### Geospatial modelling

#### Random forest modelling

To predict the occurrence probabilities of the four forest leaf types (broadleaved evergreen, broadleaved deciduous, needle-leaved evergreen and needle-leaved deciduous), we ran random forest models in Google Earth Engine^68^. In random forest, unlike traditional regression, correlation among variables does not affect model accuracy. Indeed, the ability to use many correlated predictors is one of the key benefits of machine learning models^69^. When variables are correlated, the effect of these variables is ‘shared’ across the trees in the random forest. Because random forest does not estimate coefficients as in regression, this correlation does not hinder model fit or performance, but rather complicates efforts to quantify variable importance, which is also shared across correlated variables. Thus, by including numerous variables, even if correlated, we can improve our predictive power of the model to accurately quantify current carbon.

To run the models, we set the output mode to ‘MULTIPROBABILITY’ and randomly sampled 10 individuals from each plot 100 times, weighting individuals by their basal area to model area-based leaf types. The following equation was used to transform DBH to basal area: $A=\frac{{\pi {\rm{DBH}}}^{2}}{4}$. To model individual-based leaf types, individuals were sampled without weighting them. This resulted in 100 training datasets, each containing 98,330 rows. After extracting pixel values from 58 environmental covariates, we then modelled leaf types for each training dataset with a random forest model, using the 58 environmental covariates. The correlation between our point-level response variable (leaf type) and spatially contiguous covariates then allowed us to map the global distribution of leaf types.

To train global models of forest leaf types, we first ran a grid-search procedure, exploring the results of a suite of random forest models with different hyperparameters. The hyperparameters that were allowed to vary were the number of random trees (10, 20, 50, 100 and 250), the number of variables sampled at each split (1, 2, 4, 5, 8, 10, 15, 20 and 30) and the minimum sample size at the end of the nodes (1, 2, 5, 10, 15, 20 and 30); subsampling rate was constant at 0.632. To quantify predictive accuracy, we used the Bhattacharyya coefficient to compare the predicted and observed probabilities for each pixel, as is commonly used in image processing and feature extraction^70–73^. Because four probability classes within a pixel are not independent, we cannot use standard loss functions to estimate predictive accuracy. Instead, we used the Bhattacharyya coefficient, given by ${\sum }_{i=0}^{n}\sqrt{{O}_{i}\times {P}_{i}}$, which quantifies the similarity between two vectors, O and P, with *n* categories. The Bhattacharyya coefficient falls within (0,1), equalling one only when ${P}_{i}={O}_{i}$ for all *i* within a pixel (that is, when the predictions exactly match the observed) and zero when the vectors are completely disjoint. To evaluate overall model performance, we then adopted a similar approach to that of ref. ^74^ for multinomial data^75^, using the Bhattacharyya coefficients to calculate a pseudo-*R*^2^ (${R}_{\rm{{BC}}}^{2}$) (equations 1∼5) on the basis of 10-fold cross-validation:

1 $${R}_{{\rm{BC}}}^{2}=1-\frac{{\rm{{MAE}}_{{model}}}}{{\rm{{MAE}}_{{mean}}}}$$

in which

2 $${\rm{{MAE}}_{{model}}}=\frac{\sum {E}_{i}}{N}$$

3 $${E}_{i}=1-\mathop{\sum }\limits_{i=1}^{n}\sqrt{{O}_{i}\times {P}_{i}}$$

and

4 $${\rm{{MAE}}_{{mean}}}=\frac{\sum {E}_{{i}_{{\rm{mean}}}}}{N}$$

5 $${E}_{{i}_{{\rm{mean}}}}=1-\mathop{\sum }\limits_{i=1}^{n}\sqrt{{O}_{i}\times {\bar{O}}_{i}}$$

where, MAE is the mean absolute error, *O*_*i*_ is the observed coverage of leaf type *i*, *P*_*i*_ is the predicted coverage of leaf type *i* (on out-of-fit data via cross-validation), $\bar{{O}_{i}}$ is the average coverage of leaf type *i* across all the observations and *n* is the number of forest leaf types (here, *n* = 4). Note that the summation terms in equations (3) and (5) are the Bhattacharyya coefficients between the observed multinomial distribution and the predicted distribution (equation 3) and the average distribution (equation 5). Thus, equation (3) is the predictive loss term for each pixel, with the MAE_model_ in equation (2) giving the average across all pixels, which equals zero only when the predictions perfectly match the observations. Similarly, equation (5) is the loss term for each pixel when using group-level means, with MAE_mean_ in equation (4) giving the average loss across all pixels. By comparing MAE_model_ to MAE_mean_, we follow the standard approach for computing *R*^2^ by quantifying performances relative to human predictions, with *R*^2^ = $1-{\rm{{MAE}}_{{model}}/{{MAE}}_{{mean}}}$ equalling 1 only when our predictions are perfect (MAE_model_ = 0) and *R*^2^ being $\le$0 when our predictions are equal to or worse than the mean. Importantly, as suggested in ref. ^74^, equation (1) is estimated using out-of-fit cross-validated data, where the predicted values are estimated by omitting the corresponding observed values from the training data, with the resulting pseudo-*R*^2^ used to assess our four-element model output.

To create the final maps (Fig. 2 and Supplementary Fig. 5), we used the random forest model for each training dataset with the optimal suite of hyperparameters based on the ${R}_{{\rm{BC}}}^{2}$ from the grid search. Extrapolation of our predictions across global forest areas resulted in 100 four-band global layers, with each band representing the global probability of one forest leaf type. We averaged the predictions from these 100 model outputs by taking the mean for the final map. We calculated the 95% confidence intervals across the 100 model layers^76^ to represent sampling uncertainty.

As an alternative mapping approach, we used an independent tree-based classification and regression trees (CART) model^77^ (Supplementary Fig. 15). This approach was used to test whether model performance depends on model type. If the two models (random forest and CART) show similar results, this indicates that predictions are not biased by model selection. Using the same independent ‘Tallo’ dataset^40^ used for testing the robustness of the random forest model, the CART model had an explanatory power of 0.46, which is similar to the ${R}_{{\rm{BC}}}^{2}$ of 0.47 of the random forest model. When directly comparing both models, the CART model showed 87% similarity (${R}_{{\rm{BC}}}^{2}$) with the random forest model. This suggests that our maps and predictions do not depend on the type of model, and we report the random forest model results throughout the main text since it showed slightly higher accuracy^55^.

#### Cross-validation using external data

We tested for the performance and correlation between the predictions of the area-based and individual-based random forest models. When using the same independent ‘Tallo’ dataset^40^ for testing the robustness of the random forest models, the tree-based and area-based models had an explanatory power of 46% and 47%, respectively. When directly comparing both models, the area-based model showed 89% similarity (${R}_{{\rm{BC}}}^{2}$) with the individual-based model, showing that both metrics result in similar predictions of leaf-type variation.

To further evaluate the performance of our models, we additionally compared the model predictions with satellite-derived leaf type estimates from annual land cover maps from the ESA CCI LC^41^. We used land cover layers for the years 2000, 2005, 2010 and 2015, each with a spatial resolution of 300 m × 300 m. To assign each pixel to forest leaf-type classes, we recalculated the leaf-type proportions for each layer as these represented leaf-type proportions across all ecosystem types, including grasslands. For example, we recalculated forest leaf-type proportions for pixels with 30% broadleaf deciduous forest cover, 20% needle-leaf evergreen forest cover, 10% needle-leaf deciduous forest cover and 40% non-forest cover by dividing each forest cover percentage by the total area covered by forest, resulting in 50% broadleaved deciduous, 33.3% needle-leaved evergreen and 16.7% needle-leaved deciduous. We then calculated the average values across the four years for each pixel and compared the results with our model outputs. Our area-based models explained 61% of the spatial variation in the ESA CCI LC models, with an explanatory power of 78% for broadleaved evergreen leaf-type proportions, 31% for broadleaved deciduous, 64% for needle-leaved evergreen and 19% for needle-leaved deciduous.

To generate global layers of soil features, the Soil Grids dataset relies on machine learning models informed by global, spatially explicit information on various climate variables. This global interpolation of soil information using climate data may thus lead to an overestimation of the covariation between climate and soil layers while reducing small-scale heterogeneity in soil features. To assess whether this potential caveat affects our results, we used point-level soil measurements from the WOSIS dataset, including clay content, silt content, pH and sand content. To spatially match this dataset with the full GFBi dataset containing more than 1.1 million plots across the globe^55^, we used the ‘geosphere’^78^ R package. We then selected the nearest soil observation that fell within 250 m or 1,000 m of the centre of each forest plot. This resulted in a spatial match between soil measurements and forest plots in 146 cases for the 250 m radius and in 1,893 cases for the 1,000 m radius (Supplementary Fig. 14a). To test whether model performance and predictions change when using point observations of soil features instead of global layers, we then trained random forest models using either WOSIS or Soil Grids soil data along with information on climate, topography, human and vegetation characteristics (Supplementary Fig. 14b–d). For both the 250 m and 1,000 m buffer radii, the models showed a high degree of agreement (${R}_{{\rm{BC}}}^{2}=0.99$) between model predictions.

In a second step, we then tested whether the use of point observations vs global layers of soil features affects the estimated importance of variables in driving leaf-type variation. The analysis showed a slight reduction in the importance of soil variables of 5–6% when using point observations rather than Soil Grids data (Supplementary Fig. 13), which is probably driven by the slightly lower covariation of soil point data with climate variables (Supplementary Fig. 16). Nevertheless, the results remain similar, showing that this difference is unlikely to affect the conclusions of our study.

#### Interpolation vs extrapolation in model predictions

To evaluate how well our training dataset represents the full multivariate environmental covariate space, we performed a principal-component-analysis-based approach following refs. ^76,79^. We projected the covariate composite into the same space using the centreing values, scaling values and eigenvectors from the principal component analysis of the training data. We created the convex hulls for each of the bivariate combinations from the top principal components and classified whether each pixel falls in or outside each of these convex hulls. We used 24 principal components with 276 combinations for all covariates for the sampling dataset. This analysis showed that 99.2% of land pixels (778,975,911 of 785,150,461) cover at least 90% of the environmental variables present in our training data locations (Supplementary Fig. 17).

#### LOO-CV

To account for the potential effect of spatial autocorrelation in model residuals on model validation statistics, we ran spatially buffered LOO-CV for a series of buffer radii from 10 m to 500 km. In LOO-CV, each observation is predicted on the basis of a model that includes all data outside the respective buffer radius. This results in 9,781 (=total number of observations) separate models for each buffer radius. Model performance was evaluated on the basis of ${R}_{{\rm{BC}}}^{2}$. To assess the range of spatial autocorrelation, we ran semi-variograms for random cross-validation and LOO-CV model residuals in each forest type, showing that regardless of buffer radius or validation type, our residuals show weak spatial autocorrelation (Supplementary Fig. 1). Nevertheless, when eliminating any potential effects of spatial autocorrelation on model performance by applying large buffer radii of 300 km and 500 km, the out-of-sample ${R}_{{\rm{BC}}}^{2}$ remained high (0.56 and 0.53, respectively).

### Global tree density and biomass calculation of leaf types

Tree leaf-type densities were estimated by integrating a map of the global tree density distribution^47^ with our individual-based forest leaf-type maps. For each pixel, we multiplied tree density values with modelled forest leaf-type proportions to obtain the pixel-level stem density of each leaf type. We then summed up these pixel-level abundances across the globe to estimate the total abundances of each forest leaf type. To obtain the total number of trees of each leaf type for the major forest types, we defined tropical forests as pixels falling in the biomes tropical and subtropical moist broadleaf forest (WWF^80^ biome 1), tropical and subtropical coniferous forest (biome 3) and mangroves (biome 14). Temperate forests were defined as pixels in the biomes temperate broadleaf and mixed forest (biome 4) and temperate coniferous forest (biome 5). Boreal forests were defined as pixels in the biomes boreal forest or taiga (biome 6), montane grasslands and shrublands (biome 10) and tundra (biome 11). Dry forests were defined as pixels in the biomes tropical and subtropical dry broadleaf forest (biome 2), tropical and subtropical grasslands, savannas and shrubland (biome 7), temperate grasslands, savannas and shrubland (biome 8), Mediterranean forests, woodlands and scrub (biome 12), and deserts and xeric shrubland (biome 13).

Forest biomass for each leaf type was computed by incorporating a map of global forest biomass^49^ with our area-based leaf-type models. We calculated the absolute biomass by scaling biomass density with tree canopy cover^81^ and pixel area within each pixel. This absolute biomass per pixel was then partitioned by leaf-type proportions, derived from our area-based models. By summing up the pixel-level biomass across the globe, we were able to approximate the total amount of biomass stored in each of the leaf types.

### Environmental drivers of forest leaf-type variations

To evaluate the relative importance of environmental features on forest leaf-type variation, out of the total 58 environmental covariates, we tested the effects of 29 commonly used environmental characteristics using random forest models (see Supplementary Fig. 1). We separated the environmental features into four major groups: climate, soil, topography and vegetation. To equally represent each of the three groups in the model and minimize collinearity, we selected the first six principal components from climate, soil and topography groups. These six principal components explained ≥90% of the total variation across all group variables. We included all the three vegetation characteristics, which are forest age, tree density and canopy height, into the analysis without computing the principal components. We then computed the variance inflation factors (VIFs) across all 21 principal components using the R package HH^82^. All VIFs were lower than 4, suggesting sufficient independence among covariates. The principal components were then used as predictors in random forest models with different combinations of hyperparameters (that is, 1 to 12 samples per split and a minimum sample size at the end of the nodes of 1 to 10), and we selected the ten best models with the highest coefficient of determining variation (*R*^2^). Variable importance was determined by calculating the relative influence of each variable, expressed by the variable’s attributed reduction in squared error. To quantify the importance of individual environmental factors, we used the same combinations of hyperparameters. Based on *R*^2^, the ten best random forest models were again chosen to explore the relative importance of each factor. The random forest models were run via the R package h2o^83^.

### Forest types and their future climate envelopes

To assess the extent and distribution of future changes in forest leaf-type climate envelopes, we projected our leaf-type models into the future using CMIP6 climate models for the time interval of 2071–2100 and three emission scenarios (SSP1–RCP2.6, SSP3–RCP7 and SSP5–RCP8.5)^50^. The CMIP6 climate models were extracted following the ISIMIP3b protocol and included five models (gfdl-esm4, ukesm1-0-II, mpi-esm1-2-hr, ipsl-cm6a-lr and mri-esm2-0). We projected the global forest leaf-type distribution for each emission scenario on the basis of each of the five climate models using our random forest models. To do so, we used our 100 random forest models of present leaf-type distributions and replaced the climate variables (‘bioclim’ variables from the CHELSA dataset, marked with hashtags in Supplementary Fig. 1) with CMIP6 climate model projections while keeping soil, topographic, vegetative and anthropogenic characteristics constant. For each emission scenario and CMIP6 model, we aggregated the 100 layers by taking the mean. We then aggregated the five CMIP6 model projections by taking the mean. For both evergreen vs deciduous and broadleaf vs needle-leaf proportions, we then subtracted the present predictions by the averaged model projections for the three climate change scenarios. We then summarized the predictions for each of the three climate change scenarios across latitude, aggregating predictions for each half degree. This allowed us to identify areas where future climates will shift to conditions that currently support leaf types different from the prevailing ones (Fig. 5 and Supplementary Fig. 9). To do so, we first obtained information on the forest type that currently is most abundant in each pixel (using area-based leaf-type proportions). Pixels in which > 60% of the forest area was covered by a single leaf type were assigned to that respective leaf type. Pixels in which none of the leaf types covered > 60% of the forest area were categorized as mixed forest (Fig. 5). The analysis was also conducted for a forest-area threshold of 80% to ensure that the results are not driven by the choice of the threshold (Supplementary Fig. 9). Following these definitions, we categorized forest pixels into groups using present and future climate scenarios. By scaling pixels by canopy cover, we could then calculate the total areas in which the climate is expected to shift to conditions that currently support a different forest type.

### Reporting summary

Further information on research design is available in the Nature Portfolio Reporting Summary linked to this article.

### Supplementary information

Supplementary Information Supplementary Table 1 and Figs. 1–17.

Reporting Summary

## Data Availability

Tree occurrence data from the Global Forest Biodiversity initiative (GFBi) is available upon request via Science-I (https://science-i.org) or the GFBi website (https://www.gfbiinitiative.org/). Information on leaf habit (evergreen vs deciduous) and leaf form (broadleaved vs needle-leaved) came from the TRY database (https://www.try-db.org). Additional, leaf-type data came from the Tallo dataset (https://zenodo.org/record/6637599). Plot-level soil information came from the World Soil Information Service (WOSIS) dataset (https://www.isric.org/explore/wosis).

## Code availability

All code is available at 10.5281/zenodo.7967245.

## References

[1] Pan Y, Birdsey RA, Phillips OL, Jackson RB (2013). The structure, distribution, and biomass of the world’s forests. Annu. Rev. Ecol. Evol. Syst..

[2] Pan Y (2011). A large and persistent carbon sink in the world’s forests. Science.

[3] *The State of the World’s Forests 2020. Forests, Biodiversity and People* (FAO and UNEP, 2020).

[4] Bonan GB (2008). Forests and climate change: forcings, feedbacks, and the climate benefits of forests. Science.

[5] Wright IJ (2004). The worldwide leaf economics spectrum. Nature.

[6] Schulze ED (2006). Biological control of the terrestrial carbon sink. Biogeosciences.

[7] Sayer EJ (2006). Using experimental manipulation to assess the roles of leaf litter in the functioning of forest ecosystems. Biol. Rev. Camb. Phil. Soc..

[8] Ollinger SV, Aber JD, Reich PB, Freuder RJ (2002). Interactive effects of nitrogen deposition, tropospheric ozone, elevated CO_2_ and land use history on the carbon dynamics of northern hardwood forests. Glob. Change Biol..

[9] Nicotra AB (2011). The evolution and functional significance of leaf shape in the angiosperms. Funct. Plant Biol..

[10] Díaz S (2016). The global spectrum of plant form and function. Nature.

[11] Baldocchi DD (2010). On the differential advantages of evergreenness and deciduousness in mediterranean oak woodlands: a flux perspective. Ecol. Appl..

[12] Arora VK, Boer GJ (2005). A parameterization of leaf phenology for the terrestrial ecosystem component of climate models. Glob. Change Biol..

[13] Schweitzer JA (2004). Genetically based trait in a dominant tree affects ecosystem processes. Ecol. Lett..

[14] Tian F (2018). Coupling of ecosystem-scale plant water storage and leaf phenology observed by satellite. Nat. Ecol. Evol..

[15] Méndez-Alonzo R, Pineda-García F, Paz H, Rosell JA, Olson ME (2013). Leaf phenology is associated with soil water availability and xylem traits in a tropical dry forest. Trees.

[16] Givnish TJ (2002). Adaptive significance of evergreen vs. deciduous leaves: solving the triple paradox. Silva Fenn..

[17] Axelrod DI (1966). Origin of deciduous and evergreen habits in temperate forests. Evolution.

[18] Reich PB, Walters MB, Ellsworth DS (1997). From tropics to tundra: global convergence in plant functioning. Proc. Natl Acad. Sci. USA.

[19] Villar R, Merino J (2001). Comparison of leaf construction costs in woody species with differing leaf life‐spans in contrasting ecosystems. New Phytol..

[20] Chabot BF, Hicks DJ (2003). The ecology of leaf life spans. Annu. Rev. Ecol. Syst..

[21] Augusto L (2015). Influences of evergreen gymnosperm and deciduous angiosperm tree species on the functioning of temperate and boreal forests. Biol. Rev..

[22] Flo V (2021). Climate and functional traits jointly mediate tree water-use strategies. New Phytol..

[23] Lin YS (2015). Optimal stomatal behaviour around the world. Nat. Clim. Change.

[24] Choat B (2012). Global convergence in the vulnerability of forests to drought. Nature.

[25] Lusk CH, Wright I, Reich PB (2003). Photosynthetic differences contribute to competitive advantage of evergreen angiosperm trees over evergreen conifers in productive habitats. New Phytol..

[26] Mekonnen ZA, Riley WJ, Randerson JT, Grant RF, Rogers BM (2019). Expansion of high-latitude deciduous forests driven by interactions between climate warming and fire. Nat. Plants.

[27] Baltzer JL (2021). Increasing fire and the decline of fire adapted black spruce in the boreal forest. Proc. Natl Acad. Sci. USA.

[28] Mack MC (2021). Carbon loss from boreal forest wildfires offset by increased dominance of deciduous trees. Science.

[29] Kikuzawa K (1991). A cost-benefit analysis of leaf habit and leaf longevity of trees and their geographical pattern. Am. Nat..

[30] Huechacona-Ruiz AH (2020). Mapping tree species deciduousness of tropical dry forests combining reflectance, spectral unmixing, and texture data from high-resolution imagery. Forests.

[31] Sitch S (2003). Evaluation of ecosystem dynamics, plant geography and terrestrial carbon cycling in the LPJ dynamic global vegetation model. Glob. Change Biol..

[32] Woodward FI, Williams BG (1987). Climate and plant distribution at global and local scales. Vegetatio.

[33] Smith B (2014). Implications of incorporating N cycling and N limitations on primary production in an individual-based dynamic vegetation model. Biogeosciences.

[34] Bondeau A (2007). Modelling the role of agriculture for the 20th century global terrestrial carbon balance. Glob. Change Biol..

[35] Gerten D, Schaphoff S, Haberlandt U, Lucht W, Sitch S (2004). Terrestrial vegetation and water balance - hydrological evaluation of a dynamic global vegetation model. J. Hydrol..

[36] Krinner G (2005). A dynamic global vegetation model for studies of the coupled atmosphere-biosphere system. Glob. Biogeochem. Cycles.

[37] Sato H, Itoh A, Kohyama T (2007). SEIB-DGVM: a new dynamic global vegetation model using a spatially explicit individual-based approach. Ecol. Modell..

[38] Liang J (2016). Positive biodiversity-productivity relationship predominant in global forests. Science.

[39] Kattge J (2011). TRY-a global database of plant traits. Glob. Change Biol..

[40] Jucker T (2022). Tallo: a global tree allometry and crown architecture database. Glob. Change Biol..

[41] *Land Cover Classification Gridded Maps from 1992 to Present Derived From Satellite Observations* (Copernicus Climate Change Service (C3S) Climate Data Store (CDS), accessed 24 March 2023); 10.24381/cds.006f2c9a

[42] Goldberg DE (1982). The distribution of evergreen and deciduous trees relative to soil type: an example from the Sierra Madre, Mexico, and a general model. Ecology.

[43] Reichert JM (2009). Estimation of water retention and availability in soils of Rio Grande do Sul. Rev. Bras. Cienc. Solo.

[44] Duong TTT, Penfold C, Marschner P (2012). Amending soils of different texture with six compost types: impact on soil nutrient availability, plant growth and nutrient uptake. Plant Soil.

[45] Yang J (2015). Leaf form-climate relationships on the global stage: an ensemble of characters. Glob. Ecol. Biogeogr..

[46] Allen CD (2010). A global overview of drought and heat-induced tree mortality reveals emerging climate change risks for forests. Ecol. Manage..

[47] Crowther TW (2015). Mapping tree density at a global scale. Nature.

[48] Gatti, R. C. et al. The number of tree species on Earth. *Proc. Natl Acad. Sci. USA* **119**, (2022). 10.1073/pnas.2115329119 PMC8833151 35101981

[49] Santoro, M. & Cartus, O. *ESA Biomass Climate Change Initiative (Biomass_cci): Global Datasets of Forest Above-ground Biomass for the Years 2010, 2017 and 2018 v.3* (NERC EDS Centre for Environmental Data Analysis, 2021).

[50] Karger DN (2017). Climatologies at high resolution for the earth’s land surface areas. Sci. Data.

[51] Reich PB (2022). Even modest climate change may lead to major transitions in boreal forests. Nature.

[52] Elsen PR (2022). Accelerated shifts in terrestrial life zones under rapid climate change. Glob. Change Biol..

[53] Graham RL, Turner MG, Dale VH (1990). How increasing CO_2_ and climate change affect forests. Bioscience.

[54] Keenan T, Maria Serra J, Lloret F, Ninyerola M, Sabate S (2011). Predicting the future of forests in the Mediterranean under climate change, with niche- and process-based models: CO_2_ matters!. Glob. Change Biol..

[55] Liang J (2022). Co-limitation towards lower latitudes shapes global forest diversity gradients. Nat. Ecol. Evol..

[56] Batjes NH, Ribeiro E, Van Oostrum A (2020). Standardised soil profile data to support global mapping and modelling (WoSIS snapshot 2019). Earth Syst. Sci. Data.

[57] Condit R, Engelbrecht BMJ, Pino D, Pérez R, Turnera BL (2013). Species distributions in response to individual soil nutrients and seasonal drought across a community of tropical trees. Proc. Natl Acad. Sci. USA.

[58] Álvarez-Yépiz JC (2017). Resource partitioning by evergreen and deciduous species in a tropical dry forest. Oecologia.

[59] Aerts R (1995). The advantages of being evergreen. Trends Ecol. Evol..

[60] Ouédraogo D-Y (2016). The determinants of tropical forest deciduousness: disentangling the effects of rainfall and geology in central Africa. J. Ecol..

[61] Chave J (2009). Towards a worldwide wood economics spectrum. Ecol. Lett..

[62] Simard M, Pinto N, Fisher JB, Baccini A (2011). Mapping forest canopy height globally with spaceborne lidar. J. Geophys. Res. Biogeosci..

[63] Sitch S (2015). Recent trends and drivers of regional sources and sinks of carbon dioxide. Biogeosciences.

[64] Pugh TAM (2020). Understanding the uncertainty in global forest carbon turnover. Biogeosciences.

[65] Hansen MC (2013). High-resolution global maps of 21st-century forest cover change. Science.

[66] Cayuela L, Granzow-de la Cerda Í, Albuquerque FS, Golicher DJ (2012). Taxonstand: an R package for species names standardisation in vegetation databases. Methods Ecol. Evol..

[67] Hengl T (2017). SoilGrids250m: global gridded soil information based on machine learning. PLoS ONE.

[68] Gorelick N (2017). Google Earth Engine: planetary-scale geospatial analysis for everyone. Remote Sens. Environ..

[69] Strobl C, Boulesteix AL, Kneib T, Augustin T, Zeileis A (2008). Conditional variable importance for random forests. BMC Bioinformatics.

[70] Simin C, Rongqun Z, Wenling C, Hui Y (2009). Band selection of hyperspectral images based on Bhattacharyya distance. WSEAS Trans. Inf. Sci. Appl..

[71] Ning J, Zhang L, Zhang D, Wu C (2010). Interactive image segmentation by maximal similarity based region merging. Pattern Recognit..

[72] Choi E, Lee C (2003). Feature extraction based on the Bhattacharyya distance. Pattern Recognit..

[73] El Merabet Y (2017). Maximal similarity based region classification method through local image region descriptors and Bhattacharyya coefficient-based distance: application to horizon line detection using wide-angle camera. Neurocomputing.

[74] Li J (2017). Assessing the accuracy of predictive models for numerical data: not *r* nor *r*^2^, why not? Then what?. PLoS ONE.

[75] Bhattacharyya A (1946). On a measure of divergence between two multinomial populations. Sankhyā Indian J. Stat..

[76] Ma H (2021). The global distribution and environmental drivers of aboveground versus belowground plant biomass. Nat. Ecol. Evol..

[77] Breiman, L., Friedman, J., Stone, C. J. & Olshen, R. A. *Classification Algorithms and Regression Trees* (Chapman & Hall, 1984).

[78] Hijmans, R. J. et al. Package geosphere (CRAN, 2019).

[79] van den Hoogen J (2019). Soil nematode abundance and functional group composition at a global scale. Nature.

[80] Olson DM (2001). Terrestrial ecoregions of the world: a new map of life on Earth. Bioscience.

[81] Tuanmu MN, Jetz W (2014). A global 1-km consensus land-cover product for biodiversity and ecosystem modelling. Glob. Ecol. Biogeogr..

[82] Heiberger, R. M. HH: Statistical Analysis and Data Display: Heiberger and Holland (CRAN, 2020).

[83] Erin, L. et al. h2o: R Interface for the ‘H2O’ Scalable Machine Learning Platform. R package v.3.32.0.2 (GitHub, 2020).

