# Supplementary material for: The global biogeography of tree leaf form and habit
Source: Nat Plants. 2023 Oct 23;9(11):1795–809. doi: 10.1038/s41477-023-01543-5 (PMC10654052; doi:10.1038/s41477-023-01543-5)
Supplement: Supplementary file 1 — Supplementary Table 1 and Figs. 1–17. [file 41477_2023_1543_MOESM1_ESM.pdf]

nature plants

Letter

https://doi.org/10.1038/s41477-023-01543-5

The global biogeography of tree leaf form and habit

In the format provided by the authors and unedited

Supplementary Information

Table S1. Information on the 58 selected covariate layers used to model forest leaf type proportions. Asterisks indicate variables that were used to analyze the importance of environmental variables on spatial variation of forest leaf types. Hashtags indicate variables that were replaced with future climate features for predicting changes in leaf type climate envelopes.

Variable	Variable group	Original spatial resolution	Units	Source
**Annual Mean Temperature*#**		≈1km	ºC * 10	Karger et al. [1]
**Annual Precipitation***			mm	
**Isothermality*#**			–	
**Max Temperature of Warmest Month#**			°C	
**Mean Diurnal Range***			°C	
**Mean Temperature of Coldest Quarter*#**			°C	
**Mean Temperature of Driest Quarter#**			°C	
**Mean Temperature of Warmest Quarter*#**			°C	
**Mean Temperature of Wettest Quarter#**			°C	
**Min Temperature of Coldest Month#**			°C	
**Precipitation of Coldest Quarter#**			mm	
**Precipitation of Driest Month#**			mm	
**Precipitation of Driest Quarter*#**			mm	
**Precipitation of Wettest Month#**			mm	
**Precipitation of Wettest Quarter*#**			mm	
**Precipitation Seasonality*#**			mm	
**Temperature Annual Range*#**			°C	
**Temperature Seasonality#**			°C	
**Annual mean wind speed**			m/s	Fick et al.[2]
**Canopy height***	Vegetation	≈1km	m	Simard et al.[3]
**Tree stem density***		≈1km	Stems/ha	Crowther et al.[4]
**Forest age***		≈1km	Years	Besnard et al.[5]
**Elevation (in meters)***	Topography	≈1km	m	Amatulli et al.[6]
**Aspect Cosine***			-	
**Aspect Sine***			-	
**Eastness***			-	
**Northness***			-	
**Profile curvature***			-	
**Tangential curvature**			-	
**Terrain roughness index**			-	
**Vector roughness messure**			-	
**Topographic position index**			-	
**Roughness***			–	
**Slope***			–	
**Human footprint in 2009**	Human	≈1km	-	Venter et al.[7]
**Human development percentage**		≈1km	%	Tuanmu & Jetz[8]

**Pixel area covered by cultivated and managed vegetation**		≈1km	%	
**Pixel area covered by urban areas**		≈1km	%	
**Irrigated rice area**		≈1km	km^2	Goldewijk[9]
**Irrigated other crops area**		≈1km	km^2	
**Rainfed rice area**		≈1km	km^2	
**Rainfed other crops area**		≈1km	km^2	
**Total actual irrigated area**		≈1km	km^2	
**Total rainfed area**		≈1km	km^2	
**Total rice area**		≈1km	km^2	
**Mean annual depth of the water table on the terrestrial land surface (in m below land surface)**	Geological	≈1km	m	Fan et al.[10]
**Absolute depth to bedrock***	Soil	≈250m	cm	Hengl et al.[11]
**Soil clay content (0–2 micrometer) at 0-100cm***			%	
**Soil coarse fragments volumetric at 0-100cm***			%	
**Soil sand content (50–2000 micrometer) at 0-100cm***			%	
**Soil silt content (2–50 micro meter) at 0-100cm***			%	
**Soil pH in H2O at 0-100cm***			pH*10	
**Soil nitrogen density***			cg/kg	
**Soil C:N ratio***		≈1km	−	Batjes et al.[12]
**Rangeland percentage per pixel**	Process	≈10km	%	Goldewijk et al.[13]
**Grazing percentage per pixel**			%	
**Cropland percentage per pixel**			%	
**pasture percentage per pixel**			%	

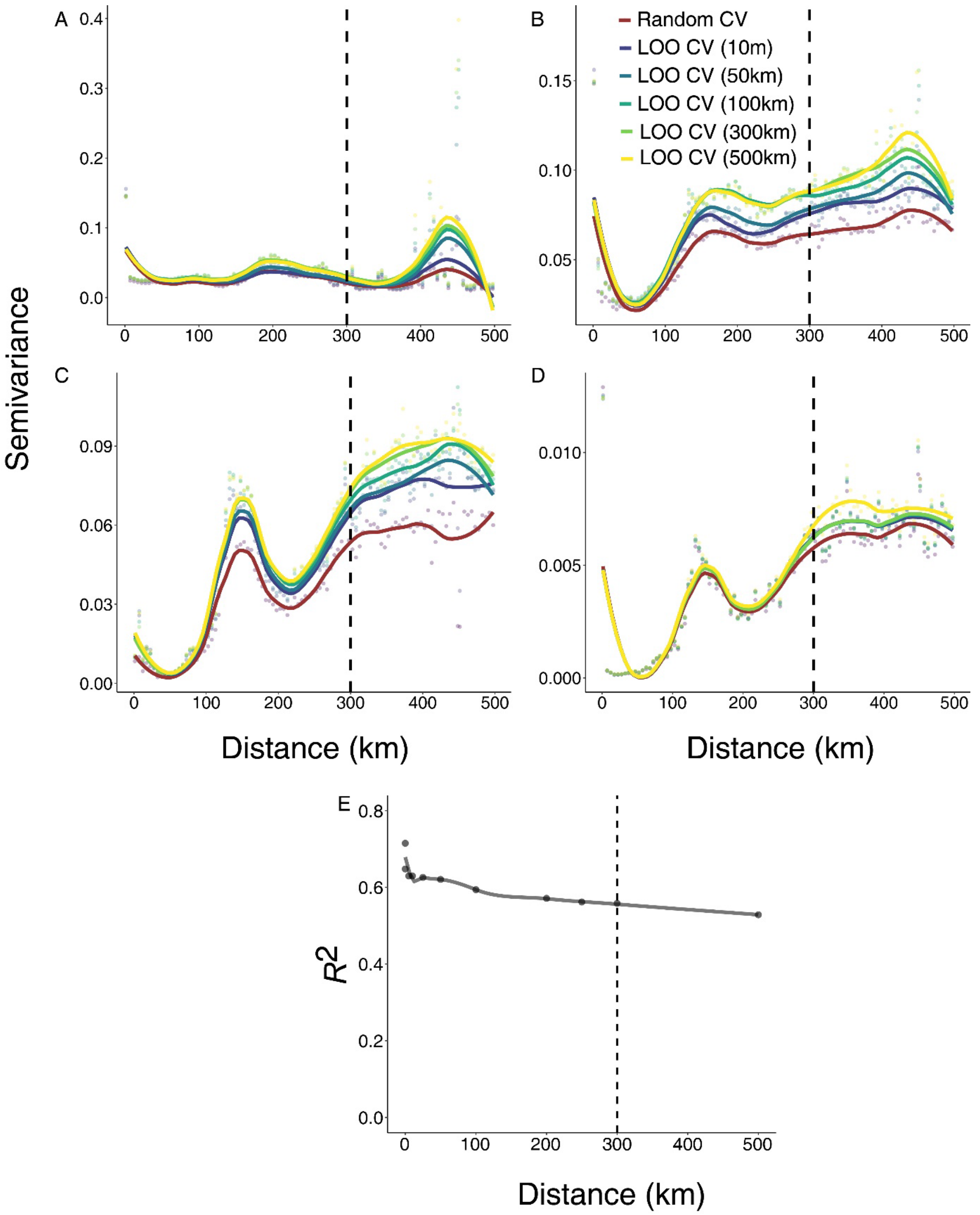

Fig. S1. Spatially-buffered leave-one-out cross validation (LOO-CV) results as semi-variograms (A-D) and model performance for varying buffer radii (E). A-D, Semi-variograms indicating the extent of spatial autocorrelation of model residuals for predictions of broadleaved evergreen (**A**), broadleaved deciduous (**B**), needle-leaved evergreen (**C**) and needle-leaved deciduous (**D**) proportions. **E**, R^2_{BC} for buffer radii of data exclusion from 10m to 500 km. Dashed lines indicate spatial buffer radii distances selected for reporting model performances.

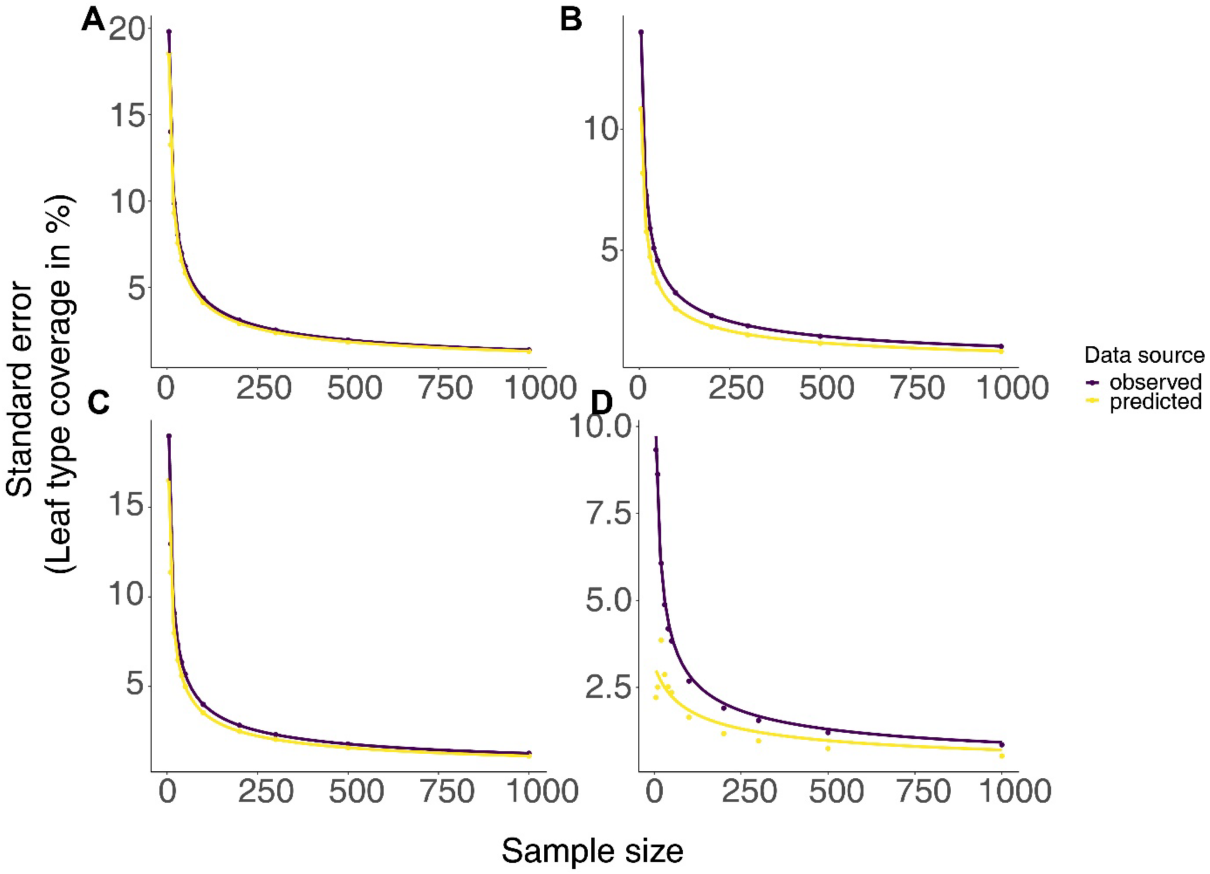

Fig. S2. Standard errors of the observed (purple) and predicted (yellow) mean values of proportions of broadleaved evergreen (A), broadleaved deciduous (B), needle-leaved evergreen (C) and needle-leaved deciduous (D) trees decrease with increasing sample size. The operation was repeated with 1,000 random seeds for the observed and predicted mean values, and the calculated standard errors of the mean are shown.

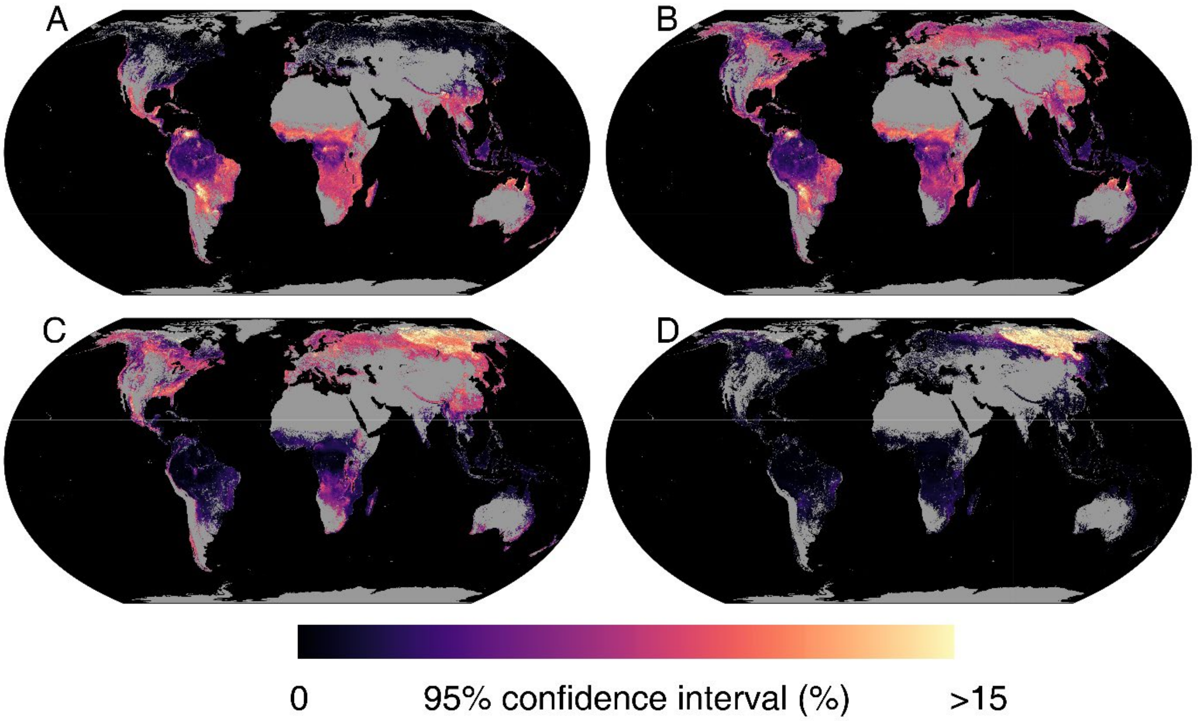

Fig. S3. Uncertainties of model predictions from random forest models for the proportions of broadleaved evergreen (A), broadleaved deciduous (B) needle-leaved evergreen (C) and needle-leaved deciduous (D) trees. For each pixel, we used the output of 100 models (differing in the sampling of individuals within plots) to calculate 95% confidence intervals as a proxy of prediction uncertainty. To calculate the relative proportion of each leaf type per plot, individuals were weighted by their basal area (area-based leaf type).

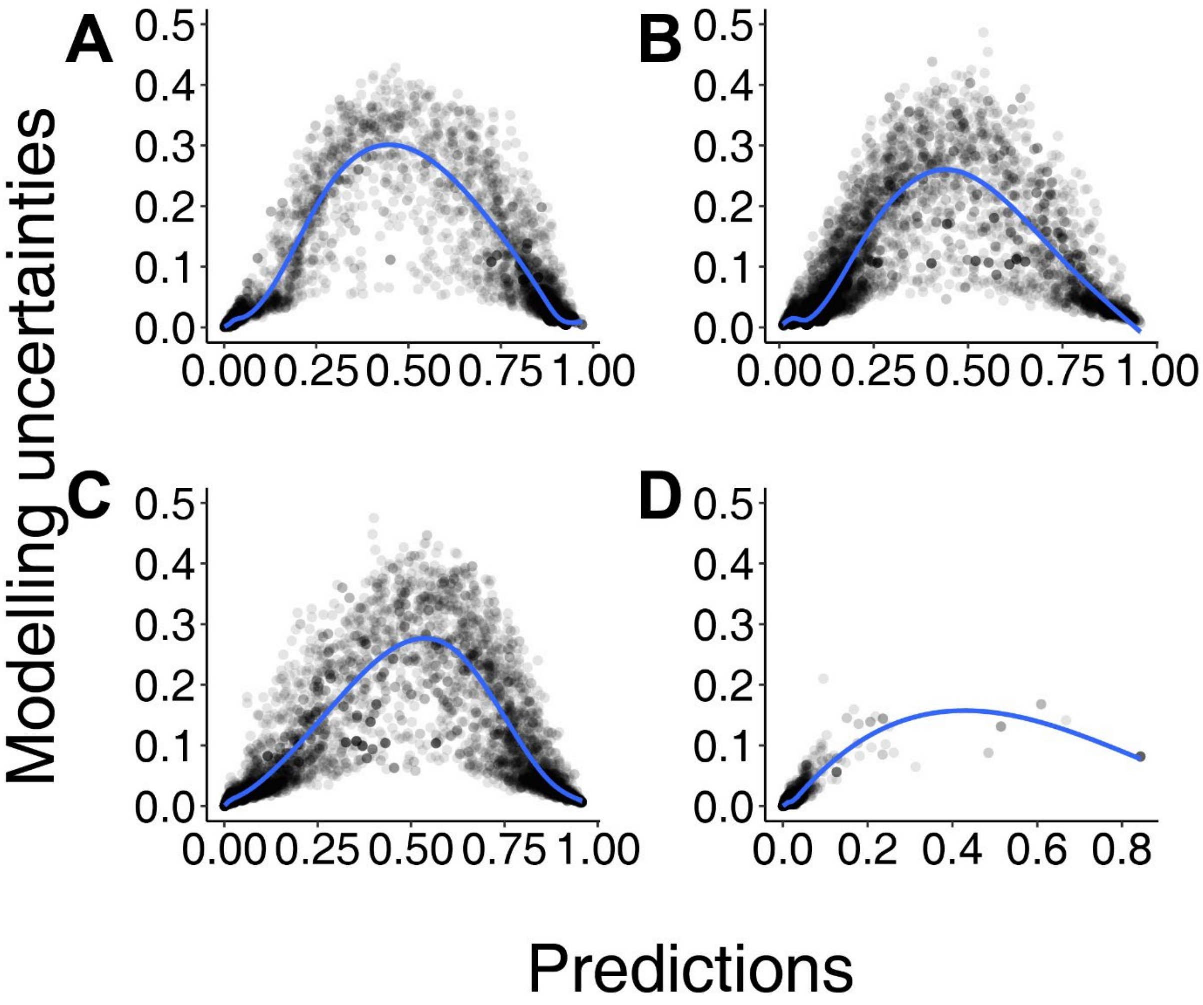

Fig. S4. Relationship between model uncertainty and model predictions for broadleaved evergreen (A), broadleaved deciduous (B), needle-leaved evergreen (C) and needle-leaved deciduous (D) forests. Modelling uncertainties (see also Fig. S3）are shown across predicted leaf-type gradients. Blue lines show the smoothed trend based on generalized additive models (GAMs). To calculate the relative proportion of each leaf type per plot, individuals were weighted by their basal area (area-based leaf type).

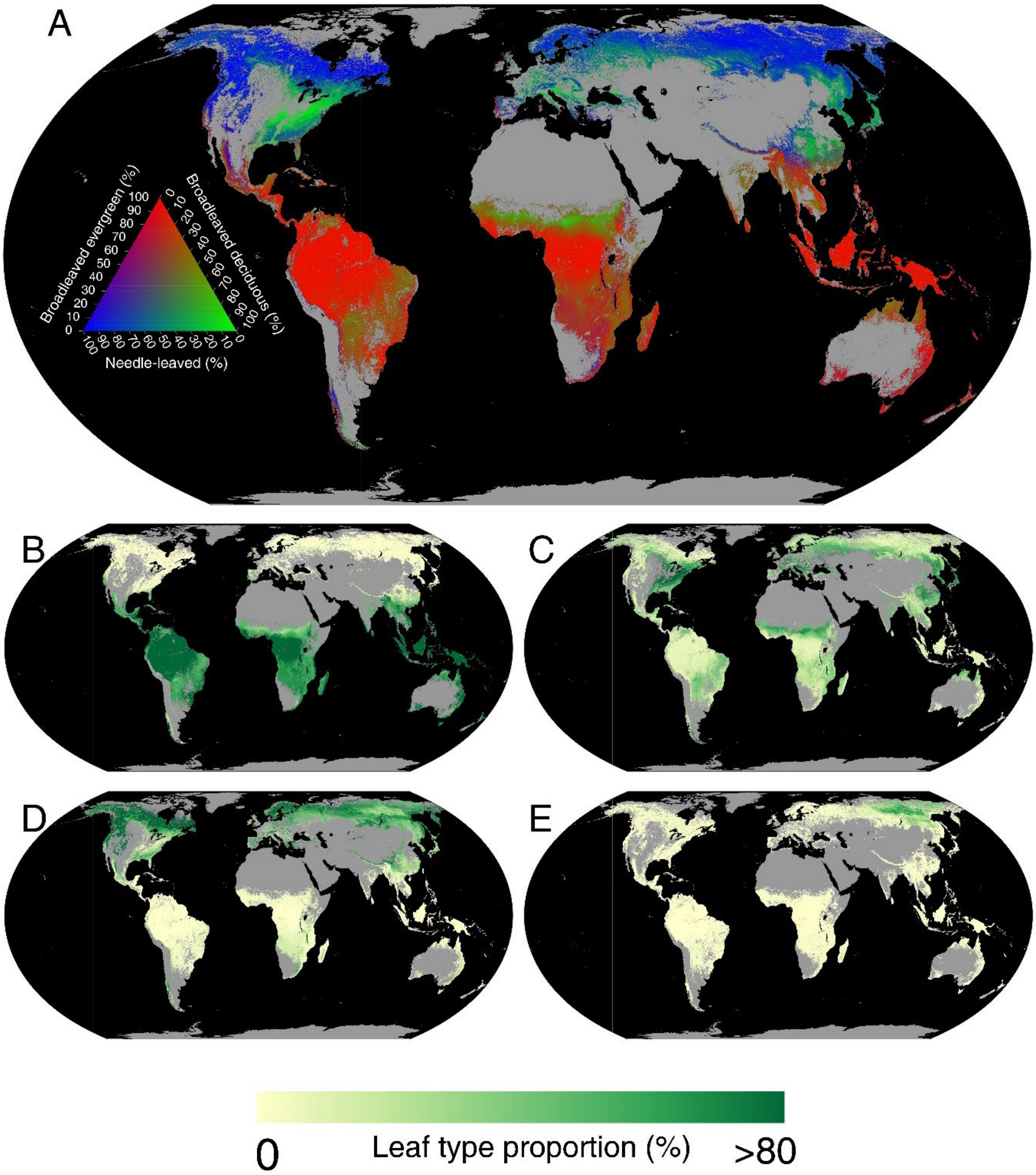

Fig. S5. The global distribution of forest leaf types (same as Fig. 2, but using individual-based leaf-type proportions instead of area-based leaf type data). A, Ternary map showing the global distribution of tree leaf type as predicted by a random forest model built from individual-based leaf-type proportions within plots (see **Methods**). Note that needle-leaved evergreen and needle-leaved deciduous forests are combined due to the low global coverage of needle-leaved deciduous trees. Red pixels represent broadleaved evergreen-dominated forests, green represents broadleaved deciduous forests and blue represents needle-leaved forests. **B**-**E**, Relative proportion of each leaf type within pixels. **B,** Broadleaved evergreen proportion. **C,** Broadleaved deciduous proportion. **D,** Needle-leaved evergreen proportion. **E,** Needle-leaved deciduous proportion.

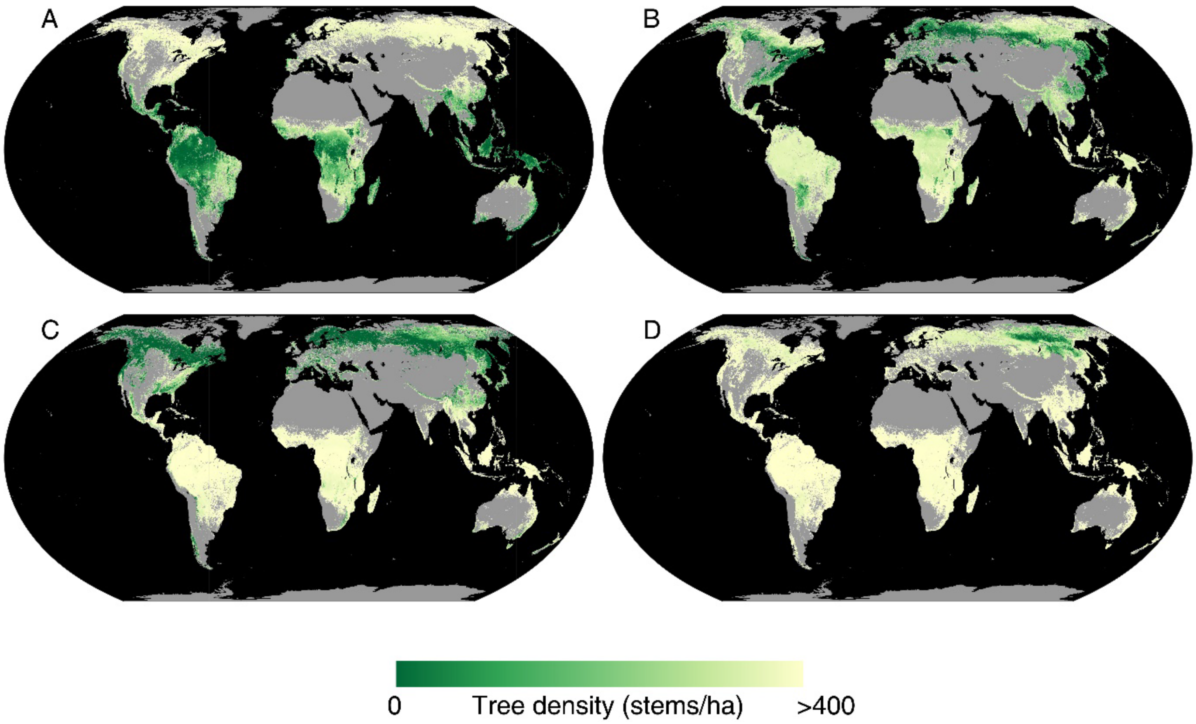

Fig. S6. The global distribution of broadleaved evergreen (A), broadleaved deciduous (B), needle-leaved evergreen (C) and needle-leaved deciduous (D) tree densities. Densities in stems per hectare.

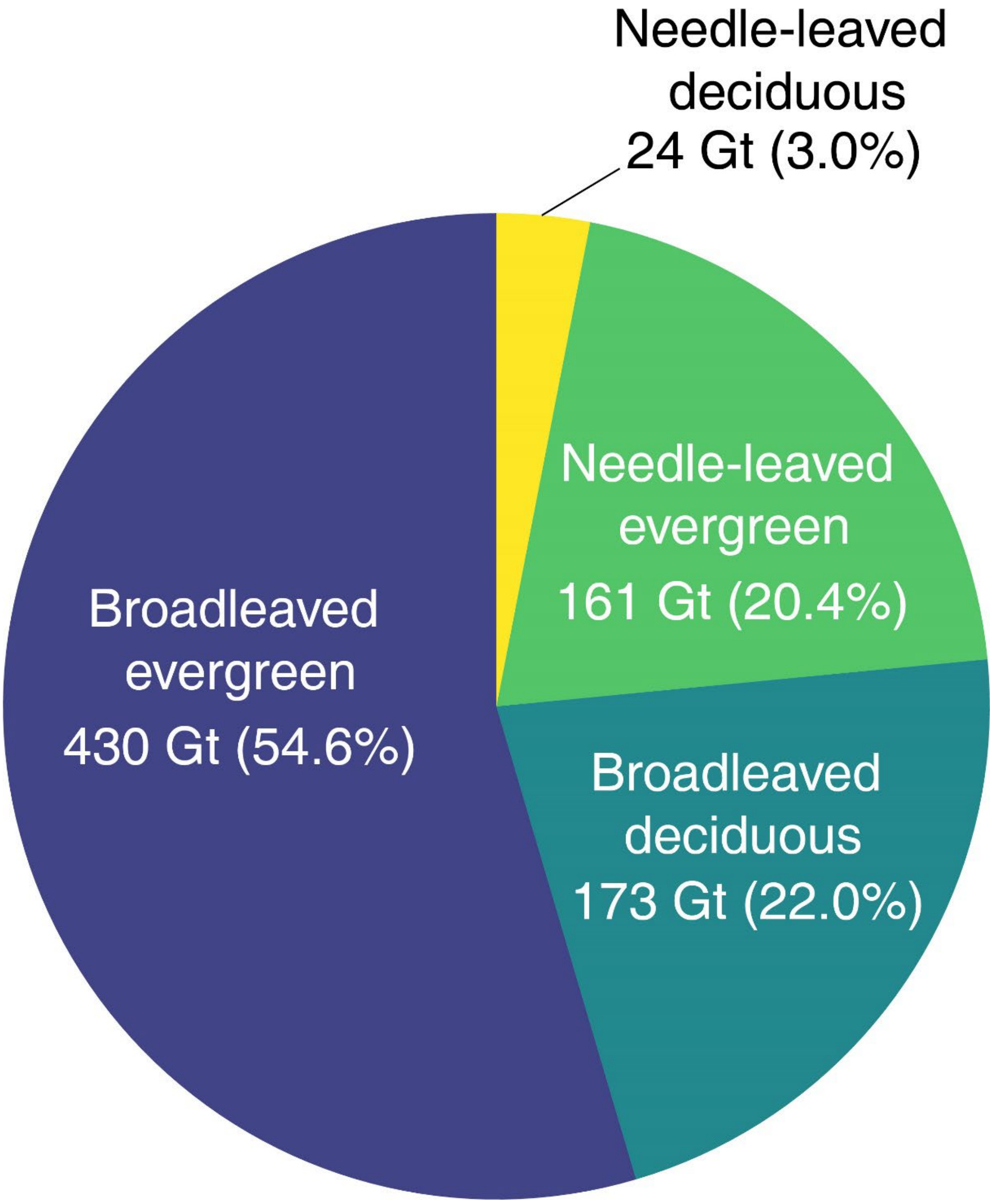

Fig. S7. The global biomass stored in broadleaved evergreen, broadleaved deciduous, needle-leaved evergreen and needle-leaved deciduous forests.

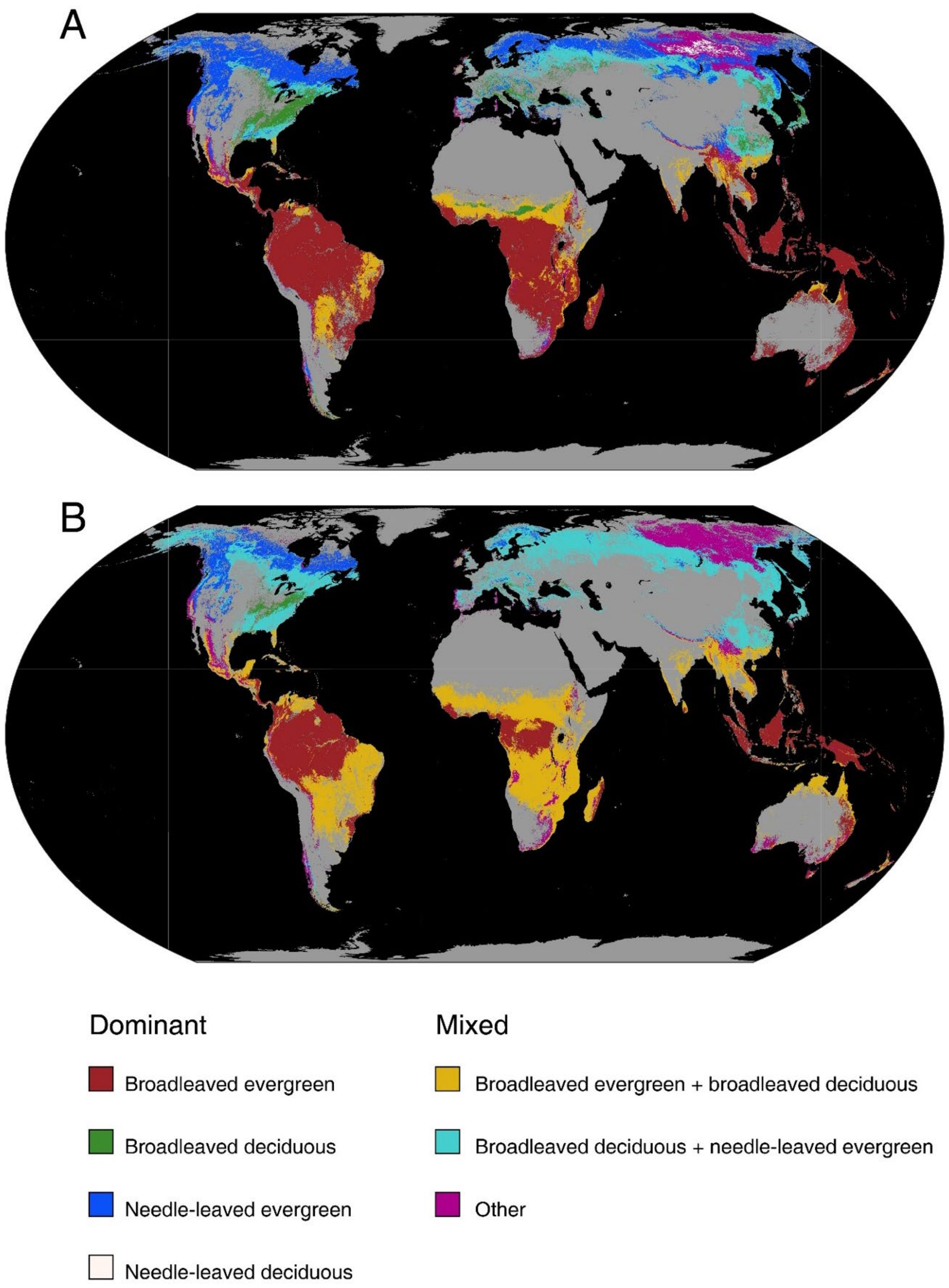

Fig. S8. Global distribution of forest types as defined by leaf-type proportions. Pixels in which >60% (**A**) or >80% (**B**) of the forest area was covered by a single leaf type were assigned to that respective leaf type. Pixels in which none of the leaf types covered more than 60% (**A**) or 80% (**B**) of the forest area were categorized as mixed forest, whereby the two main types of mixed forest (broadleaved evergreen / broadleaved deciduous and broadleaved deciduous / needle-leaved evergreen) are shown with separate colors. To calculate the relative proportion of each leaf type per plot, individuals were weighted by their basal area (area-based leaf type).

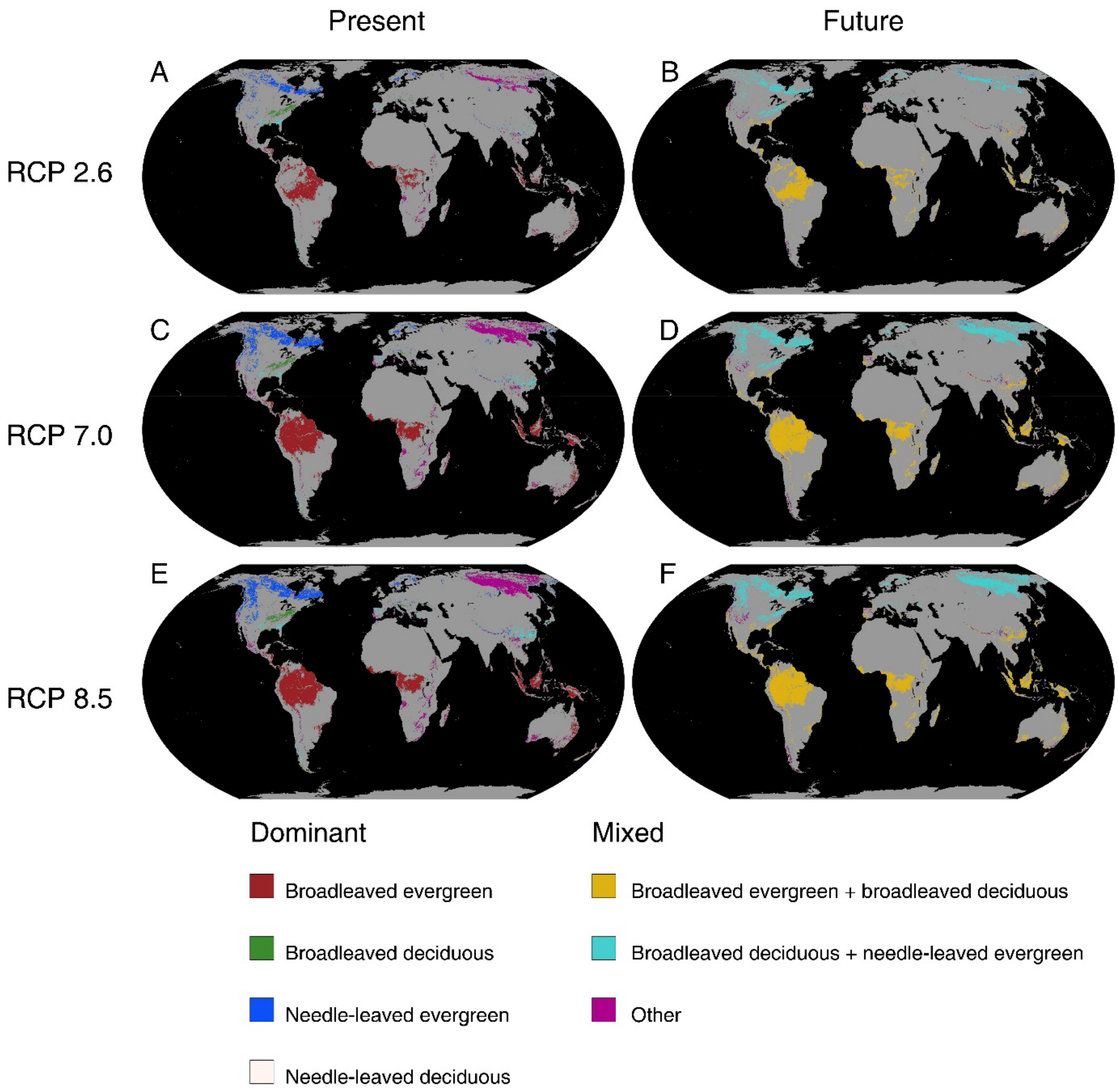

Fig. S9. Forested areas where future climates may no longer support prevailing leaf types. (same as Fig. 5 but using 80% as classification threshold of single forest types). To classify pixels into specific forest types, we established that if more than 80% of a pixel's forest area was covered by a single leaf type, it would be classified as that leaf type. Pixels where no leaf type covered more than 80% of the forest area were classified as mixed forest. To determine the relative proportion of each leaf type per plot, we considered the basal area of individual trees (area-based leaf type). Colored pixels on the map indicate areas that, by the end of the century (2071-2100), will face climate conditions that currently support a different forest type. The future climate conditions were represented using three climate change scenarios: low-emission (SSP1-RCP2.6; **A**, **B**), business-as-usual (SSP3-RCP7; **C**, **D**), and high-emission (SSP5-RCP8.5; **E**, **F**) for the period 2071–2100. Panels **A**, **C** and **E** show the present forest types, while **B**, **D** and **F** show which forest type currently exists under the future climate expected in each pixel.

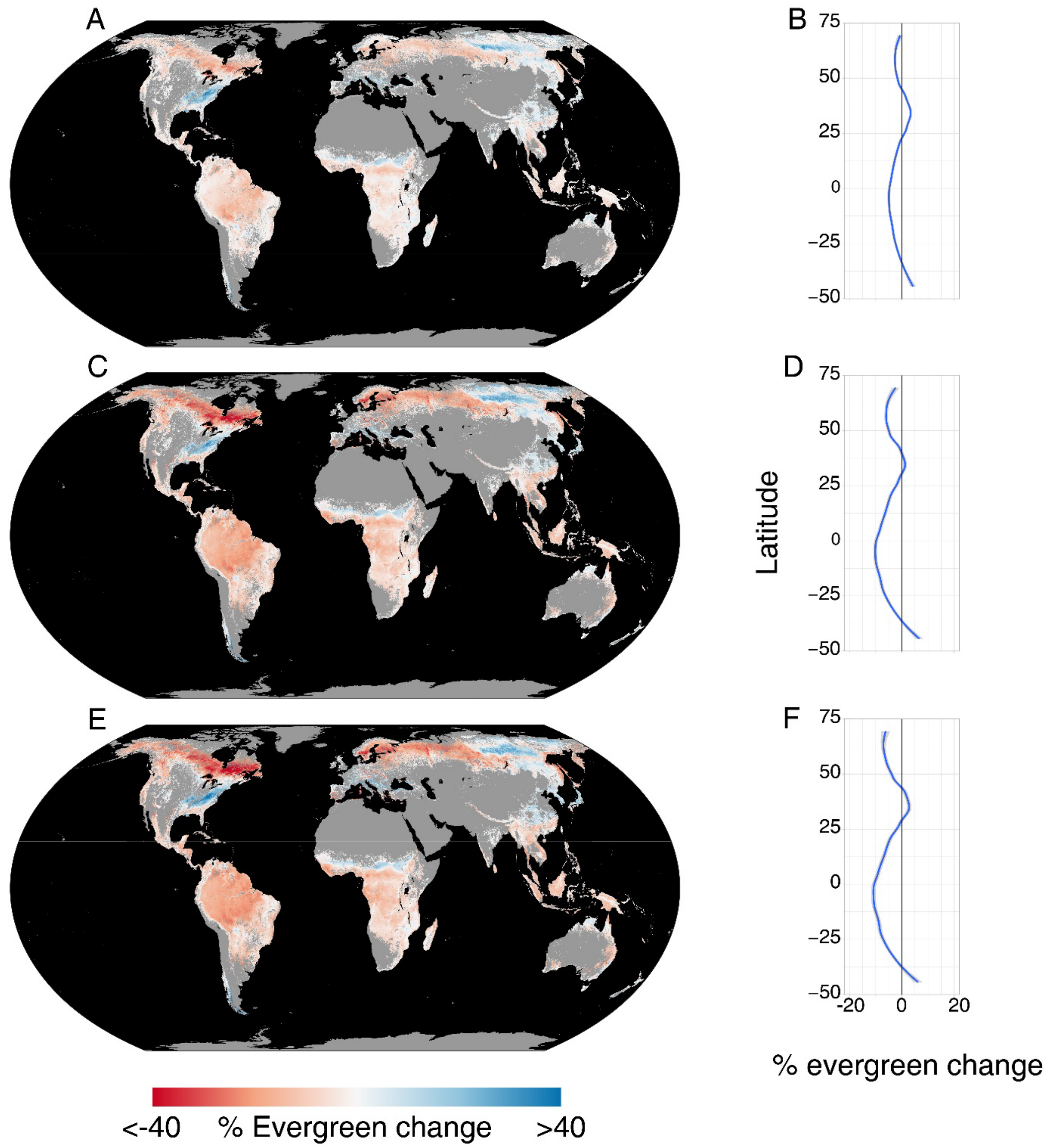

Fig. S10. Expected change in leaf-habit climate envelopes at a global scale. Climatic threat to forest leaf-type suitability, calculated as the expected climate-driven change in leaf habit (% of evergreen). To represent future climate conditions, we used low-emission (SSP1-RCP2.6; **A**), business-as-usual emission (SSP3-RCP7; **C**) and rising-emission (SSP5-RCP8.5; **E**) climate scenarios for the period 2071–2100. **B, D, F,** Latitudinal variation in the expected proportion of leaf habit under current and future (2071-2100) climate conditions in abovementioned climate change scenarios (low-emission, **B**; business-as-usual, **D** & rising-emission, **F**). Lines show mean values (solid lines) ± 1 standard error (shaded areas).

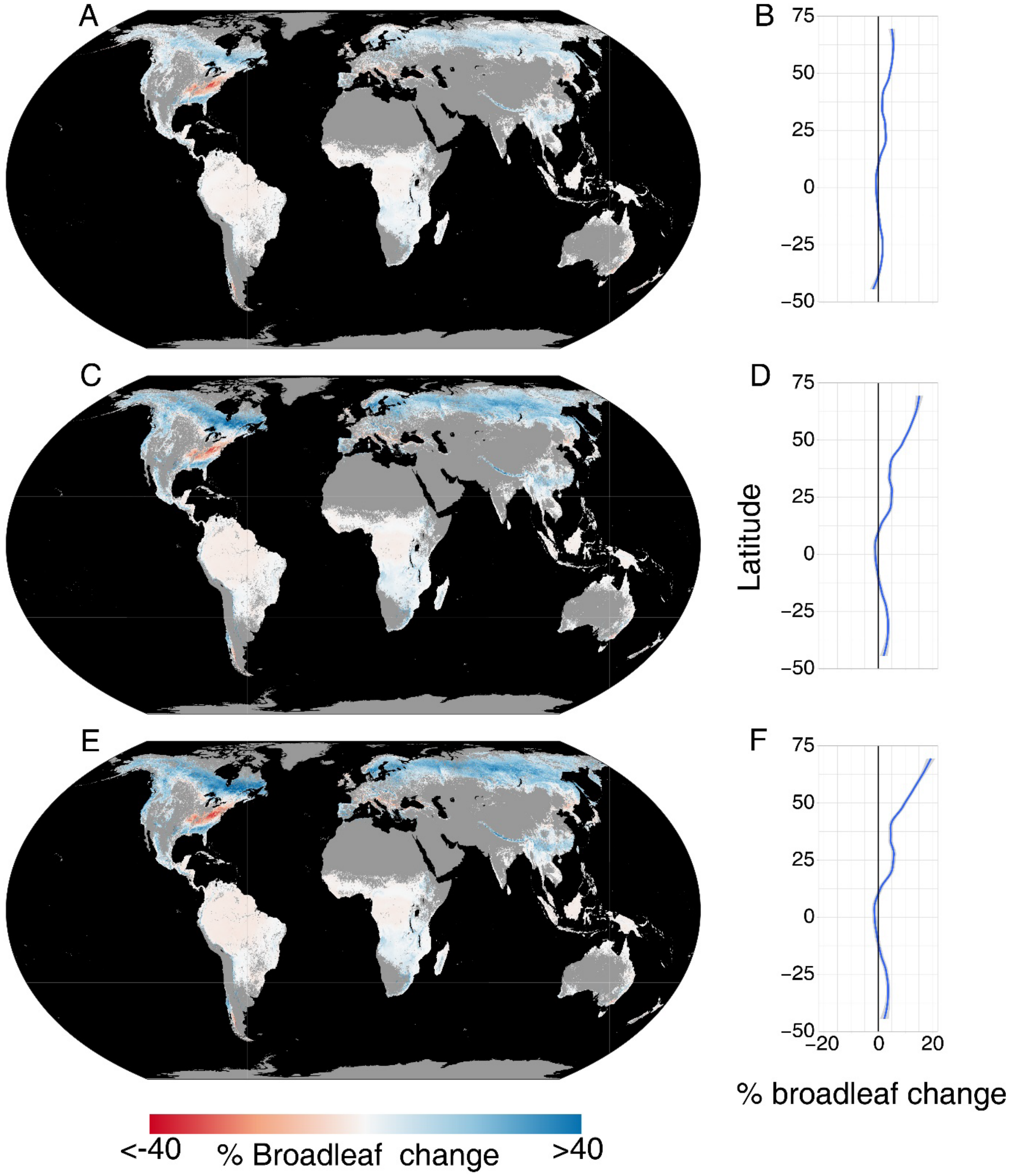

Fig. S11. Expected change in leaf form climate envelopes at a global scale. Climatic threat to forest leaf-form suitability, calculated as the expected climate-driven change in leaf form (% increase or decrease in broadleaved versus needle-leaved tree proportions). To represent future climate conditions, we used low-emission (SSP1-RCP2.6; **A, B**), business-as-usual (SSP3-RCP7; **C, D**), and high-emission (SSP5-RCP8.5; **E, F**) climate change scenarios for the period 2071–2100. **B, D & F**, Latitudinal variation in the expected change in broadleaved proportions. To calculate the relative proportion of each leaf type per plot, individuals were weighted by their basal area (area-based leaf type). Lines show mean values (solid lines) ± 1 standard error (shaded areas).

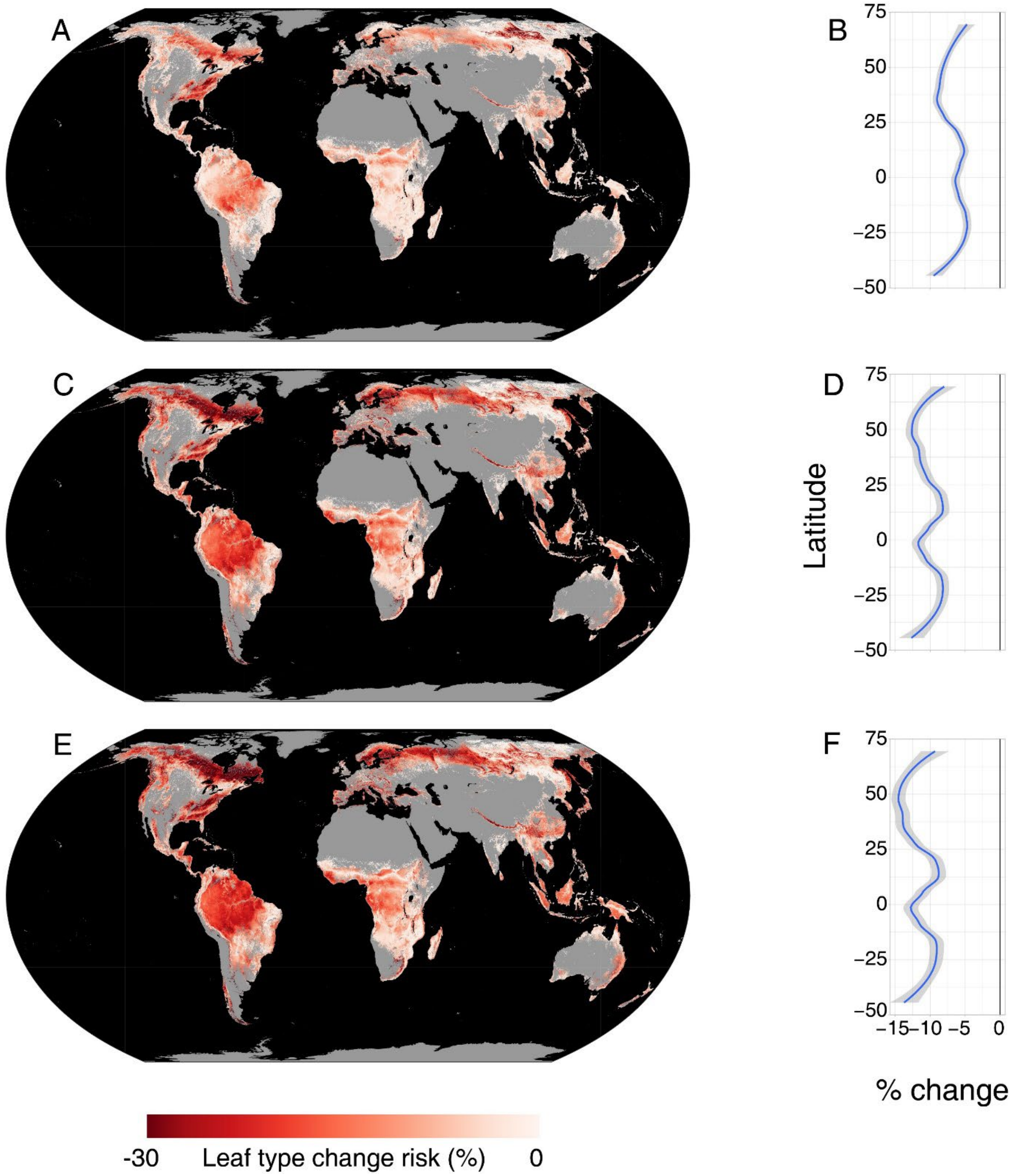

Fig. S12. **Expected changes in leaf-type climate envelopes at a global scale**. Climatic threat to forest leaf-type suitability, calculated as the expected climate-driven decrease in the leaf-type with the strongest decrease per pixel. To represent future climate conditions, we used low-emission (SSP1-RCP2.6; **A**), business-as-usual (SSP3-RCP7; **C**), and high-emission (SSP5-RCP8.5; **E**) climate change scenarios for the period 2071–2100. To calculate the relative proportion of each leaf type per plot, individuals were weighted by their basal area (area-based leaf type). **B**, **D** and **F**, Associated latitudinal variation in the expected leaf type changes. Lines show mean values (solid lines) ± 1 standard error (shaded areas).

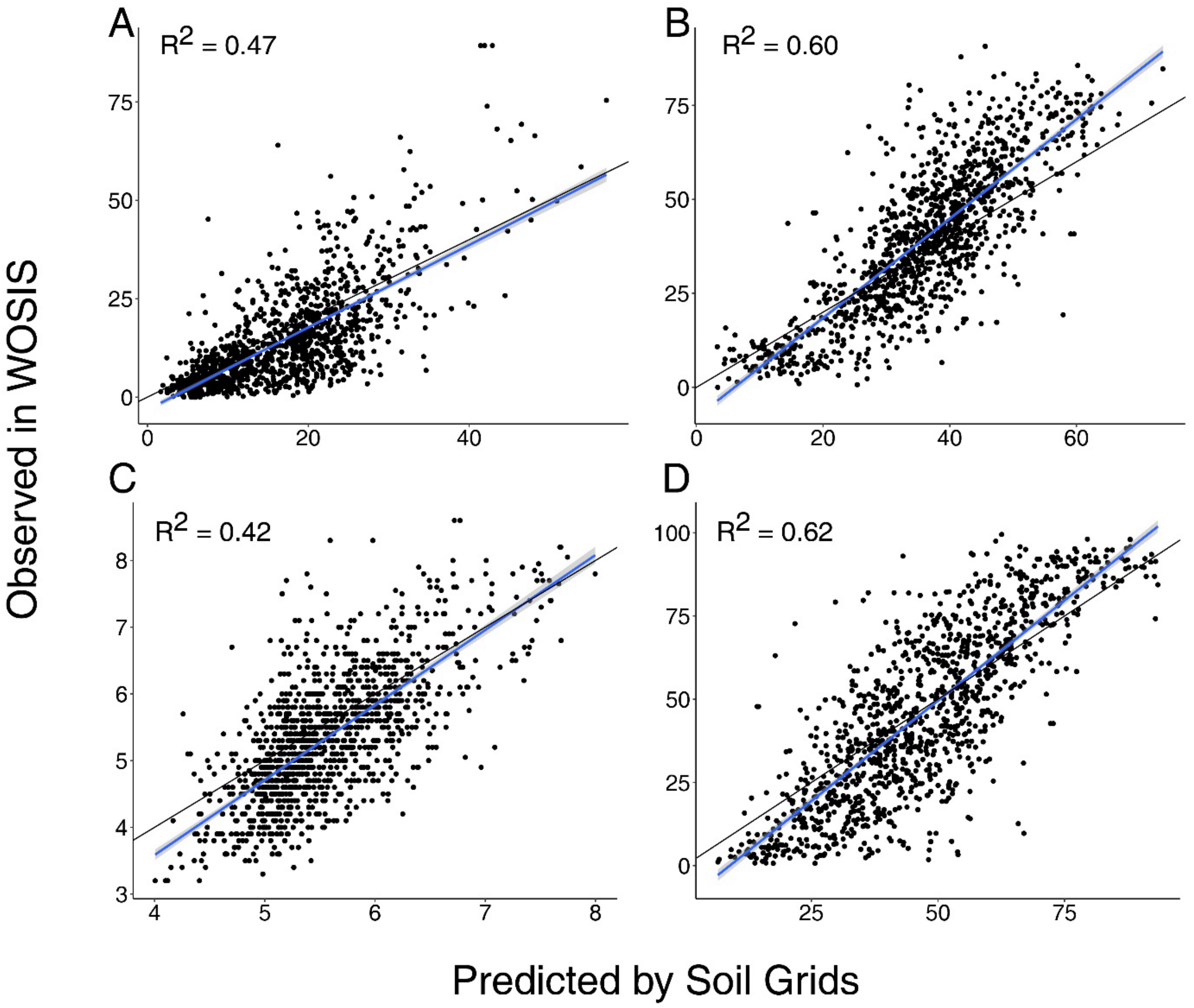

Fig. S13. Scatter plots showing the correlations of soil variables from the Soil Grids maps and the point-level WOSIS dataset. The correlations were evaluated for four variables, which were also used for forest leaf type modelling: soil clay content (**A**, mass fraction in %), soil silt content (**B**, mass fraction in %), soil pH (**C**) and sand content (**D**, mass fraction in %).

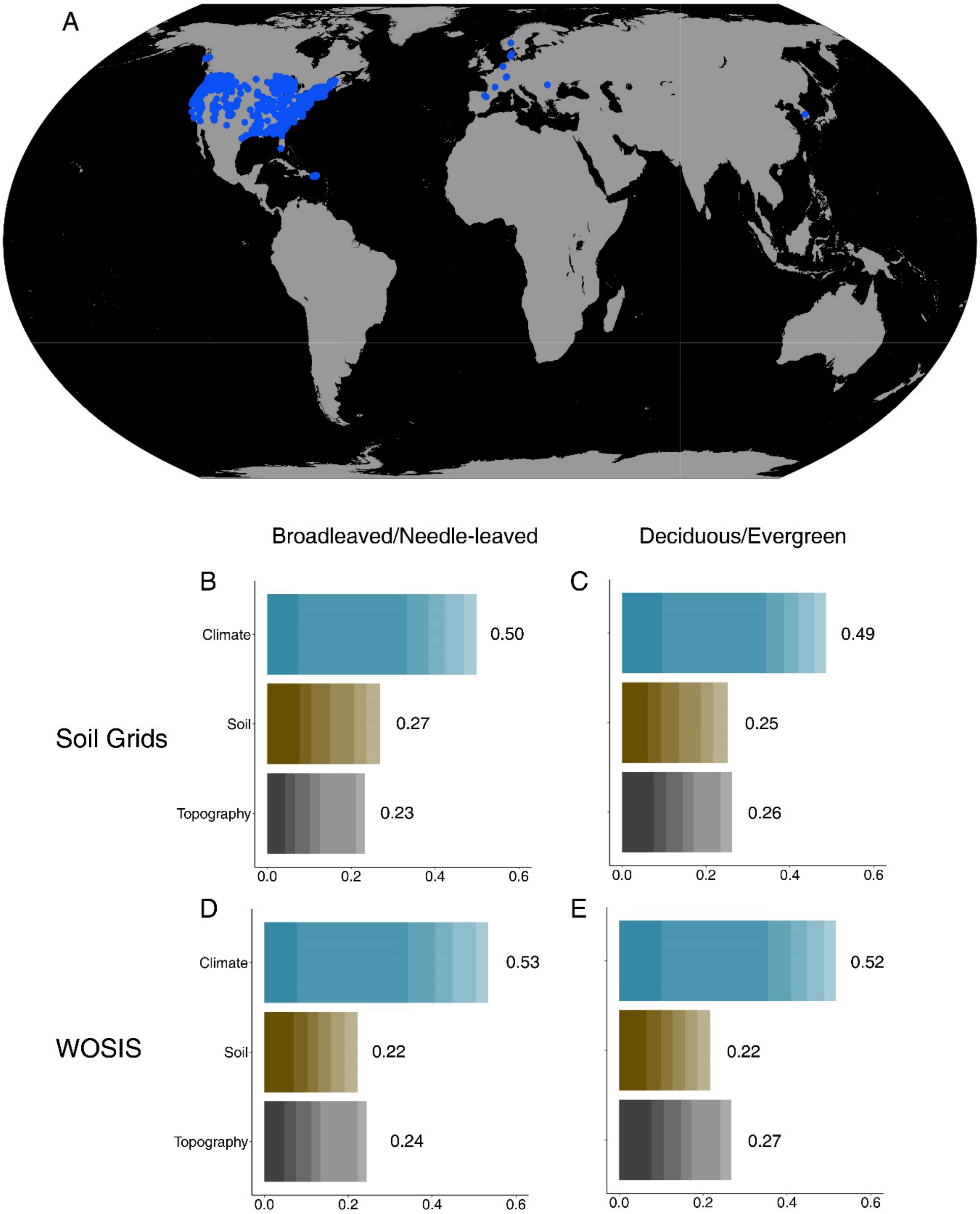

Fig. S14. Spatial distribution of sample data (A) and variable importance of environmental features on leaf type variation using gridded (Soil Grids, B & C) and point-level (WOSIS, D & E) data of soil features. Blue points in panel (**A**) represent the 1,893 locations with a match between the point-level WOSIS data and a forest inventory plot (see Method Section 2.2). Cumulative importance of the first six principal components of climate, soil and topographic covariates in the variation of leaf habit (**B, D**) and leaf form (**C, E**) with soil information coming from Soil Grids (**B, C**) or WOSIS (**D, E**).

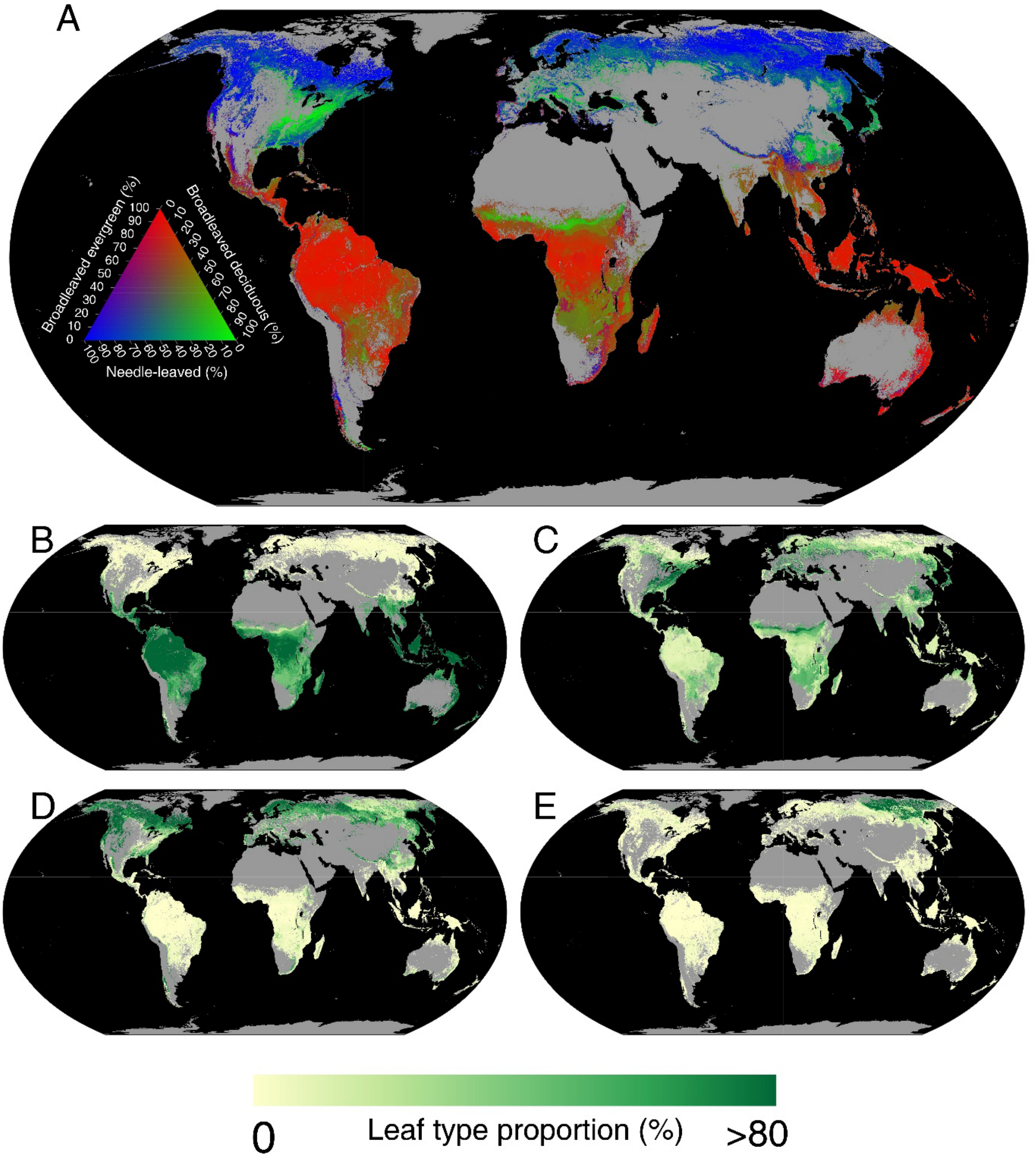

Fig. S15. The global distribution of forest leaf types (same as Fig. 2, but using a CART model instead of a random forest model). A, Ternary map showing the global distribution of tree leaf type as predicted by a CART model built from area-based leaf-type data (see Methods). Note that needle-leaved evergreen and needle-leaved deciduous forests are combined due to the low global coverage of needle-leaved deciduous trees. Red pixels represent broadleaved evergreen-dominated forests, green represents broadleaved deciduous forests and blue represents needle-leaved forests. **B**-**E**, Relative coverage of each leaf type within pixels. **B,** Broadleaved evergreen coverage. **C,** Broadleaved deciduous coverage. **D,** Needle-leaved evergreen coverage. **E,** Needle-leaved deciduous coverage.

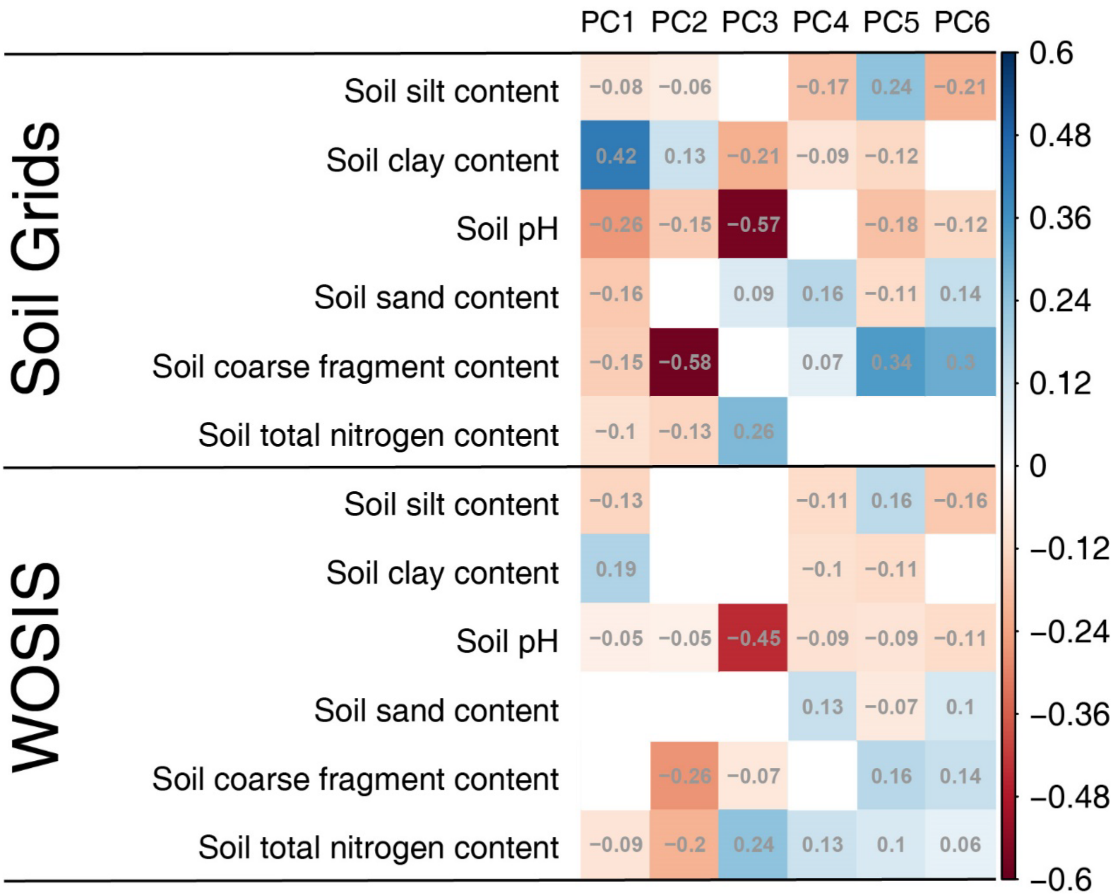

Fig. S16. Correlations between climatic principal components and soil characteristics derived from Soil Grids layers and WOSIS dataset. Colors represent magnitude and directions of correlation coefficients. Blank blocks indicate insignificant correlative relationships.

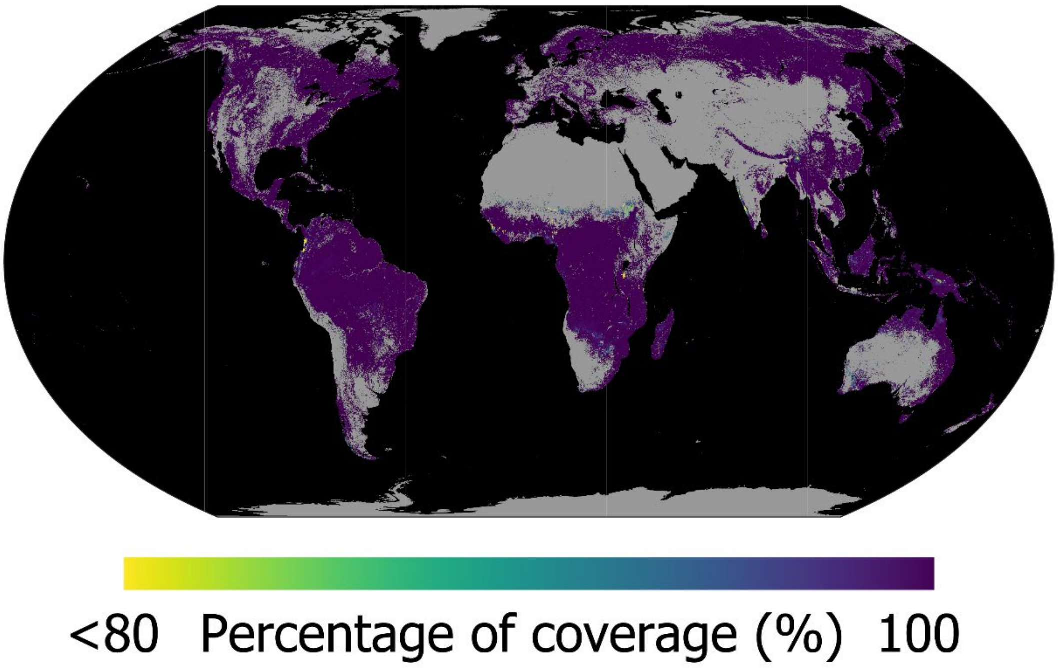

Fig. S17. The extent of interpolation and extrapolation across all forest pixels across the globe. Values represent the percentage of interpolation based on principal component analysis, that is, the percentage of bands that fall into the convex hull space.

References

1. Karger, D. N. *et al.* Climatologies at high resolution for the earth's land surface areas. *Sci. Data* **4**, 1–20 (2017).

2. Fick, S. E. & Hijmans, R. J. WorldClim 2: new 1-km spatial resolution climate surfaces for global land areas. *Int. J. Climatol.* **37**, 4302–4315 (2017).

3. Simard, M., Pinto, N., Fisher, J. B. & Baccini, A. Mapping forest canopy height globally with spaceborne lidar. *J. Geophys. Res. Biogeosciences* **116**, 4021 (2011).

4. Crowther, T. W. *et al.* Mapping tree density at a global scale. *Nature* **525**, 201–205 (2015).

5. Besnard, S. *et al.* Mapping global forest age from forest inventories, biomass and climate data. *Earth Syst. Sci. Data* **13**, 4881–4896 (2021).

6. Amatulli, G. *et al.* A suite of global, cross-scale topographic variables for environmental and biodiversity modeling. *Sci. Data* **5**, 180040 (2018).

7. Venter, O. *et al.* Global terrestrial Human Footprint maps for 1993 and 2009. *Sci. Data* **3**, 160067 (2016).

8. Tuanmu, M. N. & Jetz, W. A global 1-km consensus land-cover product for biodiversity and ecosystem modelling. *Glob. Ecol. Biogeogr.* **23**, 1031–1045 (2014).

9. Goldewijk, K. Anthropogenic land-use estimates for the Holocene; HYDE 3.2 - EASY. (2071). doi:https://doi.org/10.17026/dans-25g-gez3

10. Fan, Y., Li, H. & Miguez-Macho, G. Global patterns of groundwater table depth. *Science (80-.).* **339**, 940–943 (2013).

11. Hengl, T. *et al.* SoilGrids250m: Global gridded soil information based on machine learning. *PLoS One* **12**, e0169748 (2017).

12. Batjes, N. H. Harmonized soil property values for broad-scale modelling (WISE30sec) with estimates of global soil carbon stocks. *Geoderma* **269**, 61–68 (2016).

13. Klein Goldewijk, K., Beusen, A. & Janssen, P. Long-term dynamic modeling of global population and built-up area in a spatially explicit way: HYDE 3.1. *The Holocene* **20**, 565–573 (2010).
