# Supplementary material for: The global biogeography of tree leaf form and habit
Source: Nat Plants. 2023 Oct 23;9(11):1795–809. doi: 10.1038/s41477-023-01543-5 (PMC10654052; doi:10.1038/s41477-023-01543-5)
Supplement: Supplementary file 2 — Reporting Summary [file 41477_2023_1543_MOESM2_ESM.pdf]

nature portfolio

Corresponding author(s): Haozhi Ma

Last updated by author(s): Aug 3, 2023

Reporting Summary

Nature Portfolio wishes to improve the reproducibility of the work that we publish. This form provides structure for consistency and transparency in reporting. For further information on Nature Portfolio policies, see our Editorial Policies and the Editorial Policy Checklist.

Statistics

For all statistical analyses, confirm that the following items are present in the figure legend, table legend, main text, or Methods section.

n/a	Confirmed	
☐	☒	The exact sample size (*n*) for each experimental group/condition, given as a discrete number and unit of measurement
☒	☐	A statement on whether measurements were taken from distinct samples or whether the same sample was measured repeatedly
☒	☐	The statistical test(s) used AND whether they are one- or two-sided<br>*Only common tests should be described solely by name; describe more complex techniques in the Methods section.*
☐	☒	A description of all covariates tested
☒	☐	A description of any assumptions or corrections, such as tests of normality and adjustment for multiple comparisons
☐	☒	A full description of the statistical parameters including central tendency (e.g. means) or other basic estimates (e.g. regression coefficient) AND variation (e.g. standard deviation) or associated estimates of uncertainty (e.g. confidence intervals)
☒	☐	For null hypothesis testing, the test statistic (e.g. *F*, *t*, *r*) with confidence intervals, effect sizes, degrees of freedom and *P* value noted<br>*Give P values as exact values whenever suitable.*
☒	☐	For Bayesian analysis, information on the choice of priors and Markov chain Monte Carlo settings
☒	☐	For hierarchical and complex designs, identification of the appropriate level for tests and full reporting of outcomes
☒	☐	Estimates of effect sizes (e.g. Cohen's *d*, Pearson's *r*), indicating how they were calculated

Our web collection on statistics for biologists contains articles on many of the points above.

Software and code

Policy information about availability of computer code

Data collection	No software was used
Data analysis	The analyses were run in R (version 4.2.2) [R Core Team (2021) R: a language and environment for statistical computing. R Foundation for Statistical Computing, Vienna, Austria. http://www.R-proje ct.org. Accessed 17 Jan 2021] and Google Earth Engine (https://earthengine.google.com) (v0.1.361). The code used for this study is available at https://doi.org/10.5281/zenodo.7967245

For manuscripts utilizing custom algorithms or software that are central to the research but not yet described in published literature, software must be made available to editors and reviewers. We strongly encourage code deposition in a community repository (e.g. GitHub). See the Nature Portfolio guidelines for submitting code & software for further information.

Data

Policy information about availability of data

All manuscripts must include a data availability statement. This statement should provide the following information, where applicable:

- Accession codes, unique identifiers, or web links for publicly available datasets
- A description of any restrictions on data availability
- For clinical datasets or third party data, please ensure that the statement adheres to our policy

Tree occurrence data is available upon request from the Global Forest Biodiversity initiative (GFBi). Information on leaf habit (evergreen vs. deciduous) and leaf

form (broadleaved vs. needle-leaved) came from the TRY database. The World Soil Information Service (WOSIS) dataset contributes to the model performance evaluation. Additionally, the Tallo dataset is used to test the robustness of the random forest model.

Human research participants

Policy information about studies involving human research participants and Sex and Gender in Research.

Reporting on sex and gender	N/A
Population characteristics	N/A
Recruitment	N/A
Ethics oversight	N/A

Note that full information on the approval of the study protocol must also be provided in the manuscript.

Field-specific reporting

Please select the one below that is the best fit for your research. If you are not sure, read the appropriate sections before making your selection.

☐ Life sciences ☐ Behavioural & social sciences ☒ Ecological, evolutionary & environmental sciences

For a reference copy of the document with all sections, see nature.com/documents/nr-reporting-summary-flat.pdf

Ecological, evolutionary & environmental sciences study design

All studies must disclose on these points even when the disclosure is negative.

Study description	We analyzed the global distribution of forest leaf types, integrating ground-sourced information from 9,781 standardized forest inventory plots with leaf habit and leaf form information for a total of 10,274 species from the TRY database. Using random forest models, we quantified the global proportion and distribution of leaf types. Using information on both the occurrences of individuals per plot and the basal-area weighted occurrences of each individual, we model leaf-type proportions at the individual level (individual-based leaf type) and the basal-area level (area-based leaf type). To assess the relative importance of environmental features on variation in leaf types, we ran random forest models including a range of environmental variables. To quantify the proportions of different leaf types across global forests, we combined a global tree density distribution map with our individual-based leaf type models. To estimate biomass stored in each of the leaf types, we incorporate our area-based leaf type models with recently published forest aboveground biomass. Using the documented correlations between forest leaf types and local climate, we project the future leaf type models using three climate-change scenarios (low-emission scenario [SSP1-RCP2.6], business-as-usual scenario [SSP3-RCP7], and high-emission scenario [SSP5-RCP8.5]).
Research sample	Plot-level forest leaf types across the globe.
Sampling strategy	No statistical methods were used to predetermine sample size.
Data collection	Tree occurrence data was from Global Forest Biodiversity initiative (GFBi) dataset. Information on leaf habit (evergreen vs. deciduous) and leaf form (broadleaved vs. needle-leaved) were from TRY plant trait database.
Timing and spatial scale	The average year of observation across all plots was 2005.
Data exclusions	Individuals with stem diameters <10 cm were excluded as the focus was on adult trees, and only plots with ≥10 adult individuals were included in the final analysis. For plots with time series data, only the most recent observation year was included in the analysis.
Reproducibility	n/a
Randomization	n/a
Blinding	n/a

Did the study involve field work? ☐ Yes ☒ No

Reporting for specific materials, systems and methods

We require information from authors about some types of materials, experimental systems and methods used in many studies. Here, indicate whether each material, system or method listed is relevant to your study. If you are not sure if a list item applies to your research, read the appropriate section before selecting a response.

Materials & experimental systems

n/a	Involved in the study
☒	☐ Antibodies
☒	☐ Eukaryotic cell lines
☒	☐ Palaeontology and archaeology
☒	☐ Animals and other organisms
☒	☐ Clinical data
☒	☐ Dual use research of concern

Methods

n/a	Involved in the study
☒	☐ ChIP-seq
☒	☐ Flow cytometry
☒	☐ MRI-based neuroimaging
